# Soybean Aphid Infestation Induces Changes in Fatty Acid Metabolism in Soybean

**DOI:** 10.1371/journal.pone.0145660

**Published:** 2015-12-18

**Authors:** Charles Kanobe, Michael T. McCarville, Matthew E. O’Neal, Gregory L. Tylka, Gustavo C. MacIntosh

**Affiliations:** 1 Roy J. Carver Department of Biochemistry, Biophysics and Molecular Biology, Iowa State University, Ames, Iowa, United States of America; 2 Department of Entomology, Iowa State University, Ames, Iowa, United States of America; 3 Department of Plant Pathology and Microbiology, Iowa State University, Ames, Iowa, United States of America; University of Guelph, CANADA

## Abstract

The soybean aphid (*Aphis glycines* Matsumura) is one of the most important insect pests of soybeans in the North-central region of the US. It has been hypothesized that aphids avoid effective defenses by inhibition of jasmonate-regulated plant responses. Given the role fatty acids play in jasmonate-induced plant defenses, we analyzed the fatty acid profile of soybean leaves and seeds from aphid-infested plants. Aphid infestation reduced levels of polyunsaturated fatty acids in leaves with a concomitant increase in palmitic acid. In seeds, a reduction in polyunsaturated fatty acids was associated with an increase in stearic acid and oleic acid. Soybean plants challenged with the brown stem rot fungus or with soybean cyst nematodes did not present changes in fatty acid levels in leaves or seeds, indicating that the changes induced by aphids are not a general response to pests. One of the polyunsaturated fatty acids, linolenic acid, is the precursor of jasmonate; thus, these changes in fatty acid metabolism may be examples of “metabolic hijacking” by the aphid to avoid the induction of effective defenses. Based on the changes in fatty acid levels observed in seeds and leaves, we hypothesize that aphids potentially induce interference in the fatty acid desaturation pathway, likely reducing FAD2 and FAD6 activity that leads to a reduction in polyunsaturated fatty acids. Our data support the idea that aphids block jasmonate-dependent defenses by reduction of the hormone precursor.

## Introduction

Plants deploy biochemical and molecular strategies to deter feeding by insect herbivores. The plant hormones salicylic acid (SA), jasmonic acid (JA) and ethylene (ET) coordinate the deployment of biochemical defense against pathogens, insect pests and abiotic stresses [1]. SA is mostly involved in defense against biotrophs and hemibiotrophs [2], while JA and ET activate effective defenses against necrotrophs [3]. The response to insect herbivore attacks is mostly dependent on JA. Once plant tissues are damaged by chewing insects, linolenic acid is released from intracellular membrane lipids of the affected tissues. Linolenic acid is then processed into different oxylipins, through the octadecanoid pathway, with the ultimate production of JA [4]. JA then acts as an intracellular signal that regulates expression of defense genes. In addition, linolenic acid derivatives generated by the octadecanoid pathway can be diverted to produce green leaf volatiles that not only signal to other tissues of the injured plant or neighboring plants of an impending attack but also attract natural enemies of the herbivore [5–7].

Herbivores that feed on phloem, such as members of the insect order Hemiptera (including aphids), avoid triggering defense responses related to mechanical damage. Salivary effectors are able to inhibit occlusion of sieve elements [8]. In addition to preventing phloem sieve-element occlusion, effectors present in aphid saliva can also inhibit the deployment of effective defense responses in susceptible plants, or trigger a resistance response that results in an incompatible interaction [9]. It is hypothesized that phloem feeders avoid plant defenses by up-regulation of SA-induced responses, which in turn repress effective JA-induced responses [3, 10, 11].

The soybean aphid (*Aphis glycines* Matsumura) is a significant pest of soybean in North America that can reduce yield by up to 40% if left untreated [12]. Applications of insecticides are recommended when soybean aphid (SBA) populations reach a threshold (250 aphids per plant) to prevent yield loss [13]. Although SBA can transmit soybean viruses [14], most yield loss is from the removal of photoassimilates from phloem feeding by large populations of SBA [15]. Large populations of SBA can develop quickly, with populations doubling within as few as 2.7 days [13]. Although a significant amount of work has described the ecology of the SBA and the interactions between this insect and soybean plants at the organismal level (reviewed by [12, 16]), few reports have focused on the biochemical or molecular effects of SBA feeding on soybean plants. Microarray analysis of early changes induced by SBA feeding (6 and 12 h after infestation) identified a very rapid response in SBA-resistant soybeans carrying the *Rag1* resistance gene, and a slower response in susceptible plants, with a strong SA-dependent response observed in both cases [17]. An analysis of the interaction of SBA with soybean between 1 and 7 days after infestation showed that most resistance responses occur at the onset of infestation, while a sustained response is observed in susceptible plants up to 7 days after colonization [18]. Moreover, treatment of resistant plants with SA resulted in plants with increased resistance to SBA, indicating that, unlike the proposed scenario for Arabidopsis-aphid interactions, SA controls effective defense responses against SBA [18]. A bioinformatics analysis of the transcriptome changes induced by SBA also identified other phytohormone changes that suggest that SBA is able to trigger an abscisic acid (ABA)-dependent decoy response in susceptible plants that results in repression of JA- and SA-dependent responses [18, 19].

Metabolome analyses have also provided some insight into the metabolic changes that occur when SBA and soybean interact. Soybean plants produced a series of volatile compounds after colonization by SBA, including methyl salicylate that attracts predators of the SBA [20]. Thus, SA-dependent responses may be effective in providing direct and indirect defenses against SBA. Additional evidence suggests that the nutritional quality of plants is altered when infested with SBA [21]. In general aphid growth is limited by nitrogen [22], which is mainly provided by free amino acids in the phloem. SBA in particular are responsive to changes in the concentration of amino acids [23]. Interestingly, individual amino acid levels vary between aphid susceptible and resistant plants and these levels are also affected by aphid infestation [21]. To what extent SBA affect the quality of soybean as a host for SBA is not clear, however the evidence indicates that the soybean metabolome responds to SBA feeding.

Plant lipids are an important component of the response to aphid attacks (reviewed in [24]). Linolenic acid (18:3), a fatty acid found in membrane lipids, is the precursor for the oxylipin pathway that leads to the production of JA, and its role in defense against herbivores is well characterized [4]. Other oxylipins produced by the action of lipoxygenases and α-dioxygenases (α-DOXs) also seem to have an important role mediating effective defenses against aphids. For example, *α-DOX1*expression is induced in response to aphid colonization in Arabidopsis and tomato, and *α-DOX1* knock-down results in increased susceptibility to aphids in both plants [25]. In addition, genetic evidence indicates that other fatty acids may be important components of the response against aphids. A mutation in the Arabidopsis *SSI2* gene, which encodes a plastidyl stearoyl acyl-carrier protein desaturase that catalyzes the synthesis of oleic acid (18:1), causes enhanced resistance against *Myzus persicae* [26]. This *ssi2*-dependent resistance also requires the activity of *MYZUS PERSICAE*-INDUCED LIPASE1 (MPL1), an estearase/lipase that is induced by aphid infestation in Arabidopsis [27]. Despite the importance of fatty acids in plant-aphid interactions, the role of fatty acids in the soybean-SBA interaction is unknown.

Due to its economic importance, the interaction between soybean and SBA is an attractive model for the study of plant-aphid interactions. Our objective was to characterize the effects of SBA infestations on the fatty acid metabolism of soybeans. To determine the specificity of those effects, the impact of SBA was compared to the plant’s response to two other soybean pests, the soybean cyst nematode (*Heterodera glycines*) and brown stem rot (*Cadophora gregata*). *Heterodera glycines*, a parasitic nematode that colonizes soybean roots, is the leading yield-reducing pathogen of soybean [28]. *Cadophora gregata* is a common fungal pathogen of soybean that infects the root system and later moves to the stem, causing the brown stem rot disease and a reduction in the movement of nutrients [29]. Both *H*. *glycines* and *C*. *gregata* have been shown to undergo plant-mediated interactions with SBA when soybean is infected with all three pests [30, 31]. We also analyzed whether changes in plant fatty acid levels were observed when aphid populations were controlled following stablished management recommendations.

## Results

### Effect of aphid infestation on fatty acid composition of soybean leaves

To determine the effects of long term SBA infestation on plant defense responses in soybean, a microplot experiment was carried out in 2008 and 2009. This experiment analyzed the response of two soybean varieties (DK 27–52 and DK 28–52) to SBA infestations in a field environment. There were six different treatments: 1) aphid infestation where the population was left to develop without limit (SBA:unlimited); 2) aphid infestation where the population was left to grow to 250 aphids per plant and then sprayed with insecticide to simulate recommended management practices (SBA:250); 3) infestation of soybean plants with only the soybean cyst nematode via the soil (SCN); 4) infection with only brown stem rot fungus via the soil (BSR); 5) a combination of the SBA:unlimited, SCN, and BSR treatments (SBA:SCN:BSR); and 6) control, where plants were left untreated and free of all pests. Estimates of SBA and SCN populations and BSR disease severity ratings are shown in Table 1. These pest levels were enough to cause yield loses at the end of the two seasons ([32] and data not shown).

**Table 1:** Soybean aphid and soybean cyst nematode population densities and brown stem rot severity in 2008 and 2009 growing seasons.

	Mean SBA population density^a^ (±SE)			SCN population density^b^ (±SE)		BSR severity^c^ (±SE)	
	SBA:SCN:BSR	SBA: unlimited	SBA:250^d^	SBA:SCN:BSR	SCN	SBA:SCN:BSR	BSR
**2008**	2884±288	3983±741	553±98	1150±414	717±395	2.76±0.58	3.35±1.70
**2009**	3806.2±953	8217±1861	330±43	1570±857	2560±1091	2.10±0.32	2.30±0.44

^a^Number of SBA per plant at R3-R4 when leaf tissue samples were taken.

^b^Eggs per 100 cm^3^ soil at the end of the growing season.

^c^Number of nodes in stem displaying characteristic discoloration of brown stem rot disease.

^d^Number of SBA per plant *when insecticides were applied*. The target population was 250 aphids per plants, and was reached before the R3-R4 stage. Plants assigned this treatment were kept free of aphids throughout the rest of the experiment with additional insecticide applications as needed.

The youngest fully-developed leaf from plants exposed to each treatment were collected near the peak of aphid infestation, when plants were at late reproductive (R4-R5) growth stage, and fatty acid composition was determined. Multivariate analysis of fatty acid data for soybean leaves collected over the two seasons (2008 and 2009) revealed a significant (P<0.05) treatment effect on fatty acid levels (S1 Appendix). There was also a significant year effect but the soybean variety used had not significant effect. Treatment*year, treatment*variety, or treatment*variety*year interactions were not significant. Thus, for the leaf results described below, data for 2008 and 2009 were combined. A description of these statistical analyses is shown in S1and S2 Appendixes.

Multivariate analysis indicated that there were significant correlations among leaf fatty acid levels. In particular, palmitic acid and linolenic acid showed a strong and significant negative correlation. Palmitic acid also showed smaller but significant negative correlations with stearic and linoleic acids. A small significant positive correlation between stearic and linoleic acids was also observed. These results illustrated that the levels of individual fatty acids are not independent variables (S1 Appendix).

**Fig 1:**
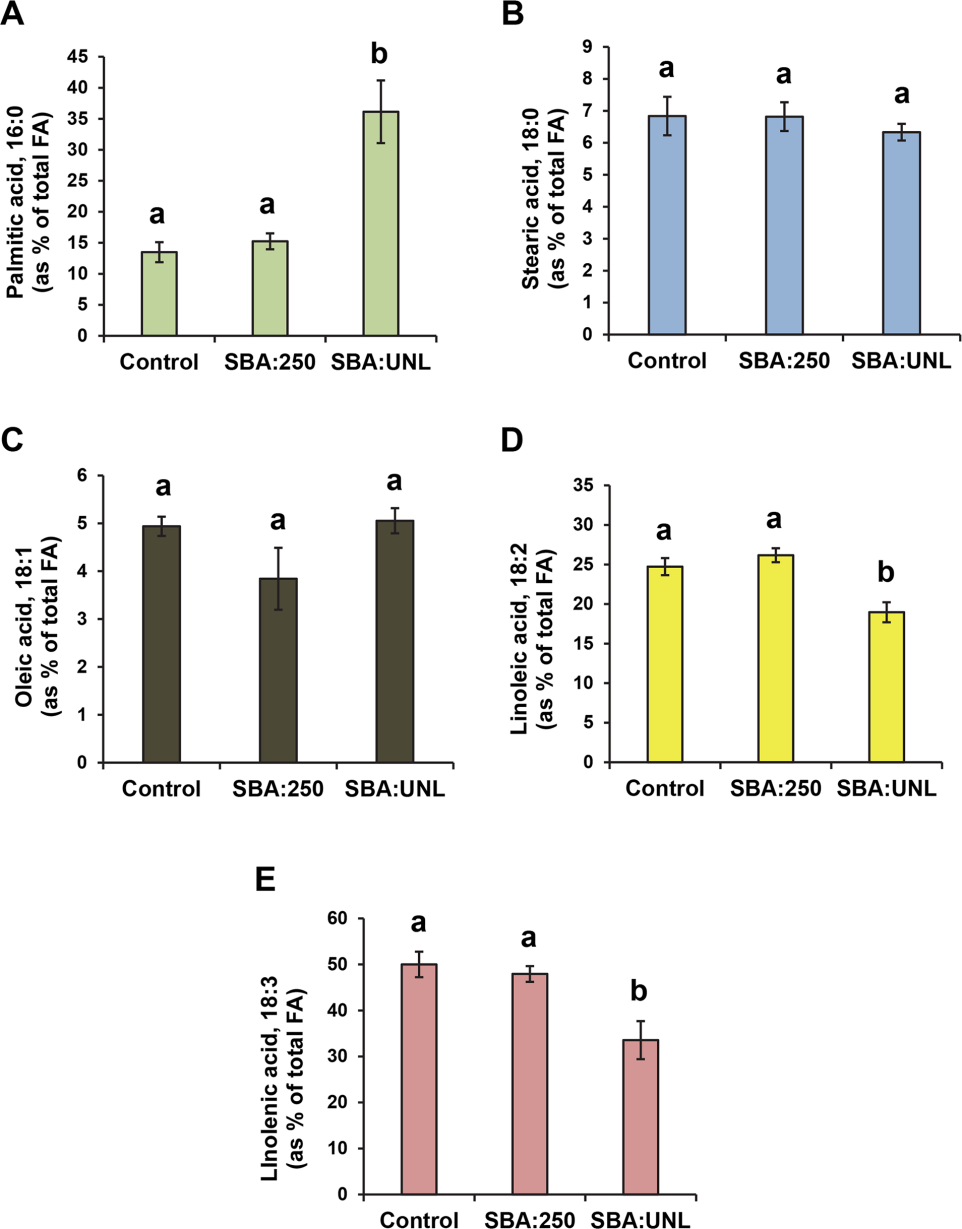
Effect of soybean aphid infestation on soybean leaf fatty acid levels. Soybean plants were infested with the soybean aphid (SBA) in 2008 and 2009. Leaves were collected six weeks after SBA infestation and fatty acid composition was analyzed. The “SBA:UNL” treatment corresponds to plants in which the aphid population was allowed to grow freely. In the “SBA: 250” treatment, plants were treated with insecticide when they reached the 250 aphids plant^-1^, as per IPM recommendations (see Material and Methods for details). Control plants were kept free of aphids throughout the whole experiment. Different letters indicate statistically significant differences (p< 0.05) among treatments.

Aphid infestation had a strong effect on the relative levels of fatty acid of soybean leaves (Fig 1 and S2 Appendix). Unlimited colonization by aphids (SBA: unlimited) resulted in a 2.7 fold increase in the level of palmitic acid in leaves (Fig 1A) compared with the level in leaves of control plants. This increase in palmitate was accompanied by a significant decrease in the levels of polyunsaturated fatty acids (PUFA) in leaves. Both linoleic (Fig 1D) and linolenic (Fig 1E) acid levels were reduced roughly 30% by unlimited aphid infestations. No significant changes were observed for stearic acid or oleic acid (Fig 1B and 1C).

We also wanted to know if plants that had been infested with aphids early in the season and then treated with insecticide, following integrated pest management (IPM) recommendations, would still show symptoms of aphid infestation at the metabolic level when seeds start developing. To address this question, plants infested with aphids were treated with insecticide when aphid populations reached 250 aphids per plant (SBA:250). These insecticide applications occurred before leaves were removed from the plant for fatty acid analysis, and these plants were kept free of aphids with additional insecticide applications as needed. Fatty acid analyses of leaves collected at the R4 stage (i.e. beginning pod fill) showed that this treatment, unlike uncontrolled aphid colonization, had no statistically significant effect on any fatty acid level (Fig 1A–1E and S2 Appendix).

**Fig 2:**
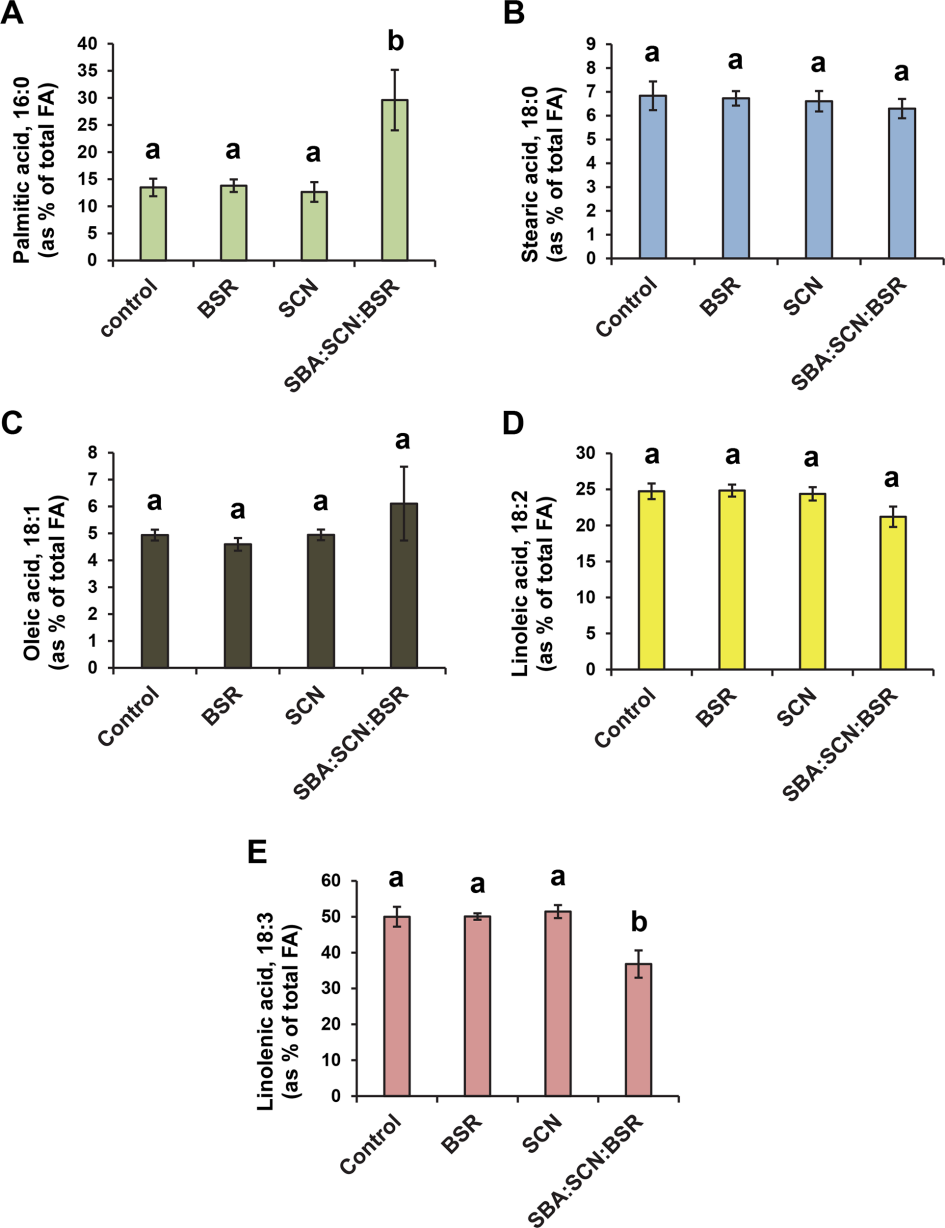
Effect of SCN and BSR fungus infections on soybean leaf fatty acid levels. For two seasons (2008 and 2009), soybean plants were infested with soybean cyst nematode (SCN), the brown stem rot (BSR) fungus or the two in combination with soybean aphid (SBA). At 6 weeks after SBA infestation, leaf samples were picked and fatty acid composition was analyzed. Control plants were kept free of aphids throughout the whole experiment. Different letters indicate statistically significant differences (p< 0.05) among treatments.

Changes in leaf fatty acid profiles could be an aphid-specific response or part of a general defense mechanism against pests. To test these possibilities, we analyzed the fatty acid profiles of plants infected with the fungal pathogen causing brown stem rot (BSR) disease or the soybean cyst nematode (SCN). Neither the BSR fungus nor SCN had a significant effect on leaf fatty acids (Fig 2A–2E).

We also analyzed the effect of a multiple pest combination (SBA:BSR:SCN) on fatty acids. There was a significant increase in palmitic acid level in leaves of plants subjected to the three treatment combination compared to the control (Fig 2A). In the three pest treatments, the level of linolenic acid significantly dropped below the control levels (Fig 2E). Since the significant differences in fatty acid content occur only when SCN and the BSR fungus are in combination with SBA, but not when SCN or the BSR fungus infect the plant individually, these differences seem to be specifically SBA-dependent.

### Effect of aphid infestation on fatty acid composition of soybean seeds

Soybean oil is an important economic resource, and its fatty acid composition has been the target of traditional breeding and biotechnology approaches aimed at improving its quality and usability for food and non-food applications [33, 34]. Since SBA were able to alter the fatty acid composition of soybean leaves, we tested whether a similar effect could be observed in seeds. Changes in fatty acid composition of grain produced from specialty soybean varieties for oil production could have important economic consequences. Thus, seeds from each treatment were subjected to fatty acid analyses after harvest. Multivariate analysis of the results showed that over the two seasons (2008 and 2009), treatments, years, varieties, and treatment*year*variety interaction significantly (P<0.05) affected fatty acid levels (S3 Appendix). This analysis also showed that there were significant correlations among seed fatty acid levels. The strongest negative correlations was observed between stearic and linoleic acids, and between oleic and linoleic acids. Smaller but significant negative correlations were also observed between stearic and linolenic acids, between oleic and linolenic acids, and between palmitic and oleic acids. Small positive correlations were observed between linoleic and linolenic acids, and between stearic and oleic acids (S3 appendix).

**Fig 3:**
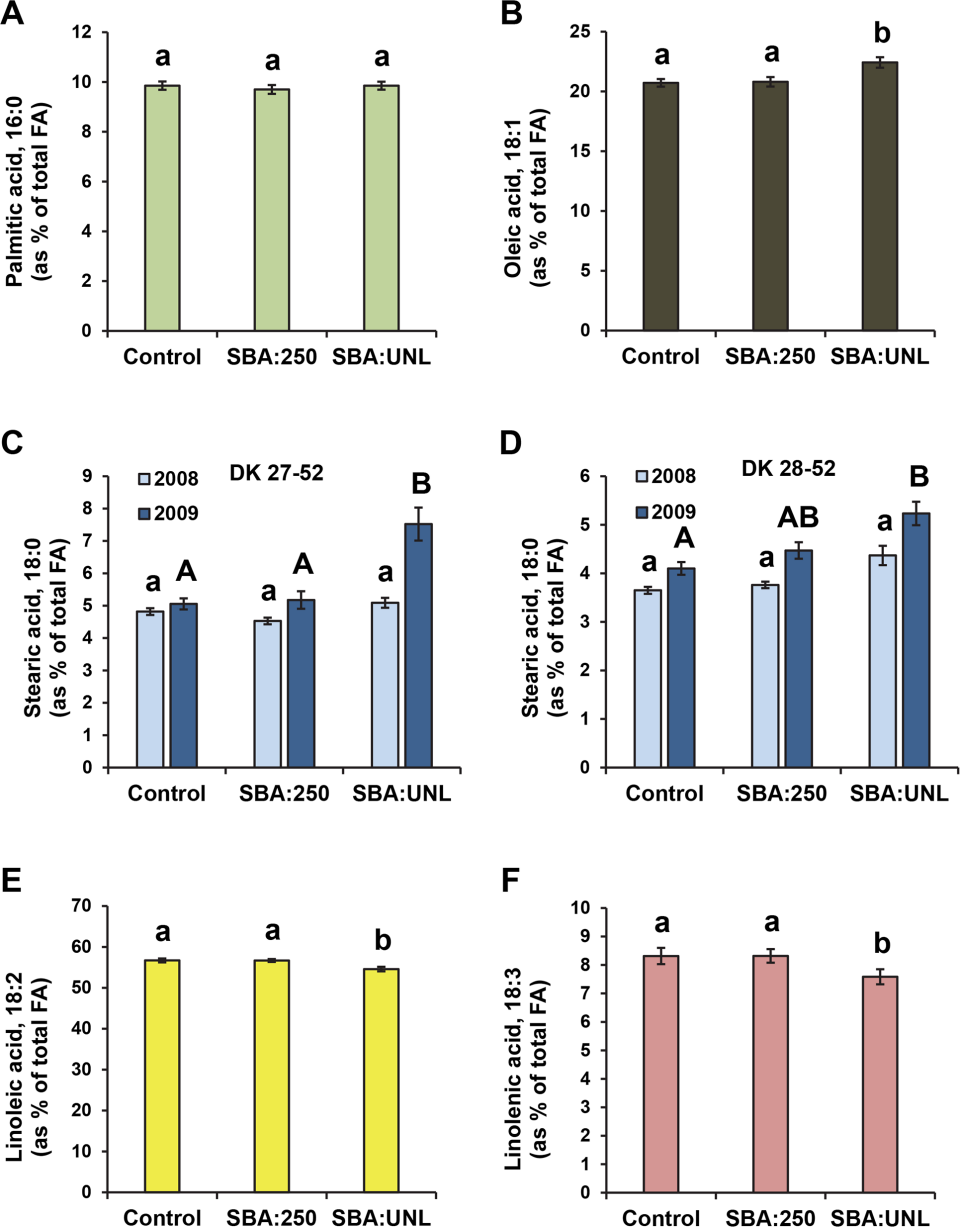
Effect of aphid infestation on fatty acids levels in soybean seeds. Seeds from soybean plants challenged with the soybean aphid were collected at the end of each of two seasons (2008 and 2009), and fatty acid analysis was carried out. Statistical analysis indicated a significant treatment*year*variety interaction only for stearic acid levels. Thus, for 18:0 (C-D), results are shown for both years and varieties separately; for other fatty acids (A, 16:0; B, 18:1; E, 18:2; F, 18:3) the results from both years and varieties were combined. Different letters indicate statistically significant differences (p< 0.05) among treatments.

Unlimited aphid infestation resulted in a significant decrease in the levels of linoleic and linolenic acids in seeds similar to the effect observed in leaves (Fig 3E and 3F and S4 Appendix), although the magnitude of the change was smaller. This decrease in PUFA was accompanied by a significant increase in the levels of oleic acid in seeds (Fig 3B). This is in contrast to the increase in palmitate observed in leaves (Fig 1). Significant changes in seed stearic acid accumulation were only observed for 2009 samples (Fig 3C and 3D and S4 Appendix). Seeds from plants subjected to recommended aphid management (SBA: 250) did not differ from the controls in the amount of any of the fatty acids analyzed (Fig 3A–3F and S4 Appendix).

**Fig 4:**
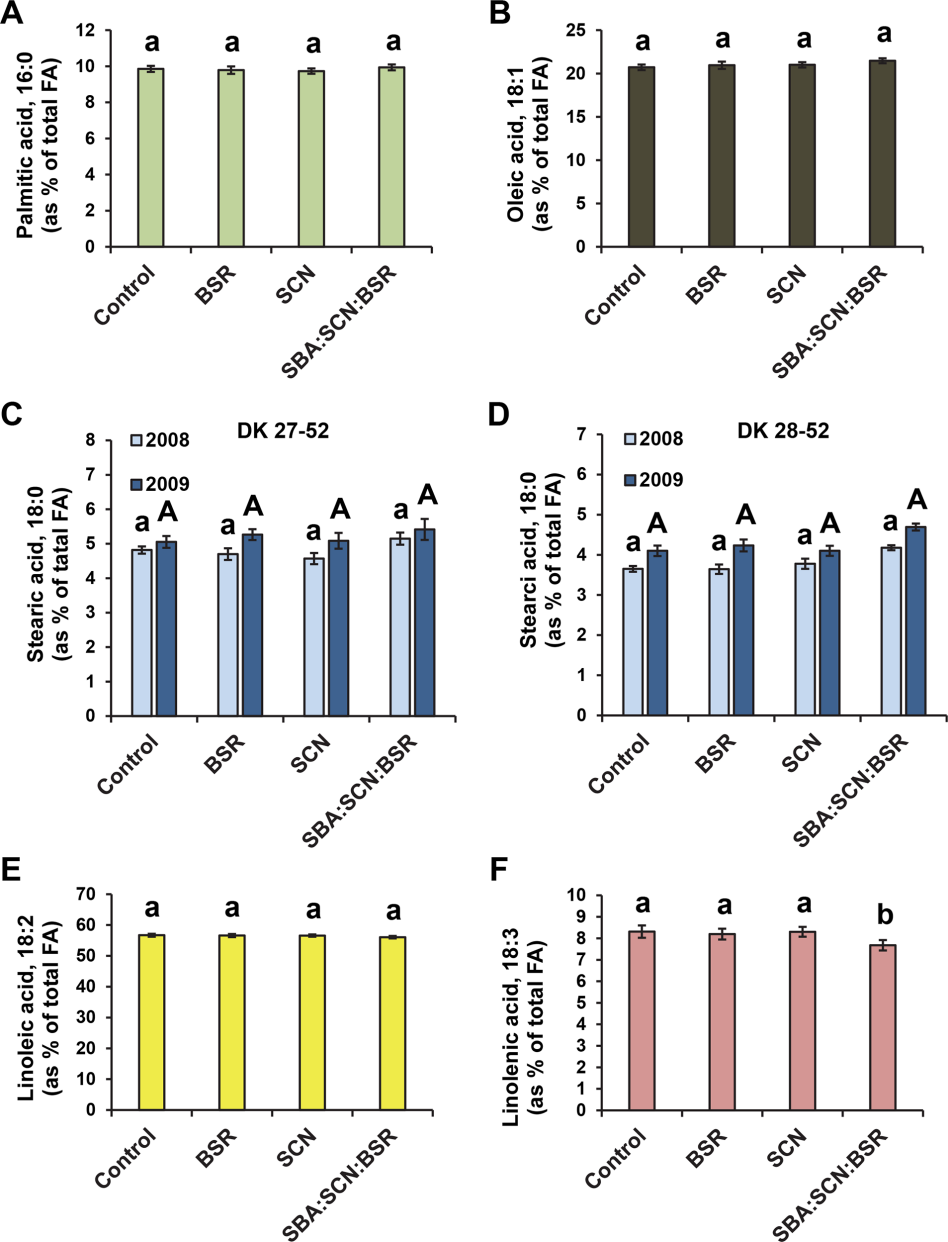
Effect of SCN and BSR fungus infections on fatty acid levels in soybean seeds. For two seasons (2008 and 2009), soybean plants were challenged with soybean cyst nematode (SCN), the brown stem rot (BSR) fungus, or the two in combination with soybean aphid (SBA). Seeds were then collected at harvest and fatty acid analysis was carried out. Statistical analysis indicated a significant treatment*year*variety interaction only for stearic acid levels. Thus, for 18:0 (C-D), results are shown for both years and varieties separately; for other fatty acids (A, 16:0; B, 18:1; E, 18:2; F, 18:3) the results from both years and varieties were combined. Different letters indicate statistically significant differences (p< 0.05) among treatments.

Root colonization by SCN and BSR fungus infections had no significant effect on the composition of seed fatty acids (Fig 4 and S4 Appendix). The combination of the three pests resulted in a significant decrease in linolenic acid content (Fig 4F) as observed in the seeds from plants infested with unlimited SBA alone. However, no significant effects were observed in linoleic acid, oleic acid, or stearic acid levels with the three pest combination (Fig 4B–4E). This result suggests that the presence of SCN, the BSR fungus or both can reduce the effects of SBA on seed fatty acid composition.

### Correlations between reduction in PUFAs and increase in saturated and monounsaturated fatty acids

Our analysis of fatty acid composition revealed that palmitic acid in the leaves increased significantly when the plants were infested with aphids (Fig 1A) while the PUFAs (linolenic and linolenic acids) decreased (Fig 1D and 1E, respectively). PUFA synthesis in leaves occurs mainly in the chloroplast, and it could be expected that a block in PUFA production in leaves results in accumulation of palmitic acid. On the other hand, we found that the decrease in PUFAs in seeds is accompanied by increases in stearic acid and oleic acid when unlimited aphids were present on the plants (Fig 3). Seed PUFAs are synthesized primarily in microsomes, and it is unclear how aphids feeding on leaves could affect this process. As a first step to understand this interaction, we carried out a correlation analysis to determine whether changes in either stearic or oleic acid could account for changes in seed PUFAs.

**Fig 5:**
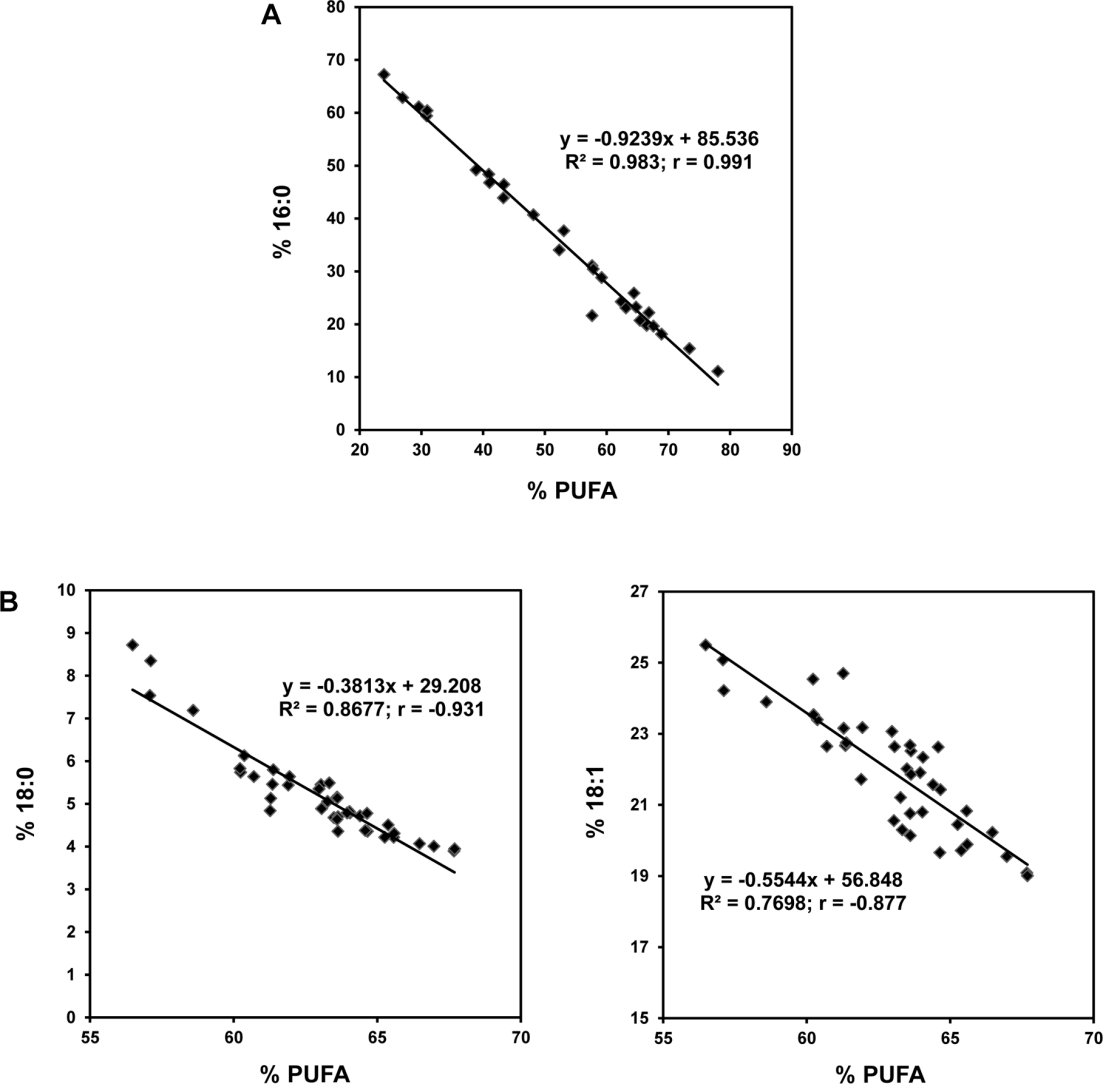
Correlations between reduction in PUFAs and increase in saturated and monounsaturated fatty acids. Strong negative correlation was observed between levels of 16:0 and PUFAs soybean leaves (A) and between levels of 18:0 and PUFAs and 18:1 and PUFAs in soybean seeds (B). PUFA levels correspond to the added percentage for 18:2 and 18:3 in each case.

As expected, a correlation of PUFA content versus palmitic acid in the leaves revealed a very strong, negative relationship with a correlation coefficient of -0.99 (Fig 5A). This gives a coefficient of determination of 98%, which is the percentage of variation in palmitic acid that is accounted for by changes in PUFAs.

In seeds, there was a strong negative correlation between PUFAs and both stearic acid and oleic acid. Correlation of PUFAs with oleic acid was consistently observed each year and showed a coefficient of -0.88 (Fig 5B), with 77% of the variation in oleic acid accounted for by the variation in PUFA. Although the aphid-induced change in stearic acid was only significant in 2009, the correlation between stearic acid and PUFAs for combined data for the two years showed a coefficient of -0.93 (Fig 5B). Moreover, when data were analyzed separately for each year, strong correlations were observed for both years, with coefficients of -0.849 and -0.966 for 2008 and 2009, respectively (S1 Fig). These results indicated that 87% of the variation in stearic acid and 77% of the variation in oleic acid could be accounted for by the variation in PUFAs.

Based on these results, it is possible to hypothesize that, following aphid infestation, a block in the production of linoleic and linolenic acids results in the increase in palmitic acid in leaves; while a block in the same pathway results in an increase in stearic and oleic acids in seeds.

## Discussion

Fatty acids are a group of biomolecules that play important roles in all living organisms. They are major components of cellular membranes, where they confer fluidity and selective permeability, and they also serve as a source of reserve energy [35]. Free fatty acids are also involved in plant responses to biotic and abiotic stress and act as alarm signals during insect attack. Certain defense responses against aphids depend on fatty acids or derived molecules (reviewed by [24]); however, no studies on the effect of SBA on soybean fatty acids have been reported to date.

In this study, we found that long-term aphid infestation results in a significant reduction in the amount of PUFAs in the leaves of soybean. The reduction in relative levels of PUFAs after long-term aphid colonization is different from the effects observed in other plant-insect interactions. For example, a generalist tobacco budworm caterpillar (*Heliothis virescens*) feeding on tall goldenrod, *Solidago altissima*, caused an increase in the levels of linoleic and linolenic acid in damaged leaves compared to the undamaged controls [36]; while mechanical wounding increases the amount of linolenic acid in tomato leaves [37]. Further demonstrating the importance of polyunsaturated acids in plant-insect interactions, an Arabidopsis mutant (*fad3-2 fad7-2 fad8*) defective in linolenic acid production was highly susceptible to the larvae of the fungus gnat *Bradysia impatiens* compared to the wild type plants that contained normal linolenic acid levels [38]. While wild type Arabidopsis plants were largely undamaged by the gnat larvae, the same treatment resulted in up to 80% mortality for the triple mutant plants. Wild type plants had a 20-fold increase in JA levels after wounding; however, wounded and unwounded mutant plants had almost undetectable levels of the hormone. The linolenic acid- and JA-deficient mutant was also associated with limited or no induction of *DHS1*, *PAL1* and *AtVSP*, genes involved in plant defense and wound response [38]. Taken together, these results highlight the importance of the interaction between PUFAs, JA and plant defense proteins in the plant response to insect attack.

The role of polyunsaturated fatty acids as precursors of JA and other octadecanoid derivatives involved in plant response to herbivory is well established [39, 40]. Once plants are damaged mechanically or by chewing insects, linolenic acid is released from membrane lipids and fed into the octadecanoid pathway, leading to the release of JA and expression of defense genes against insect attack [41]. In addition, linoleic and linolenic acids are also fed to the hydroperoxide lyase (HPL) branch of the oxylipin pathway that produces green leaf volatiles, which can have direct and indirect negative effects on insects [7, 42]. The need for linolenic acid to feed the octadecanoid pathway may explain the increase in linolenic acid levels in plant tissues damaged by insects. The same PUFAs can also be oxidized by α-DOXs that likely results in products with antibiosis effect against aphids [25].

The reduction in the proportion of unsaturated fatty acids detected in aphid-infested soybean plants may indicate that aphids are able to prevent the deployment of effective plant defenses through the octadecanoid pathway. Thus, by limiting the amount of precursor that leads to jasmonate, and/or the substrates for α-DOXs, SBA enhance their survival on soybean. Also, by reducing the amount of linoleic and linolenic acid available for the HPL pathway, aphids could limit the plants’ ability to produce volatile compounds that would not only adversely affect their performance directly [43] but also attract aphid predators and parasitoids [44].

The soybean cyst nematode is an obligated plant endoparasite that spends a large part of its life cycle in close contact with soybean. Nematode larvae penetrate the plant’s root and migrate towards the vascular bundle, where they induce the formation of a specialized feeding site, or syncytium, within or near the vascular tissue [45]. During the early stages of infection the plant starts to mount a defense response that likely utilizes JA, and SA signaling [19, 46]. However, SCN is able to locally suppress plant defenses and manipulate plant development and metabolism to establish successful feeding sites [47, 48]. The brown stem root fungus infesct plants in early seedling stages and stays latent for several weeks before transitioning to a pathogenic stage. Disease symptoms, including leaf abscission, chlorosis and necrosis of leaves, and internal browning of vascular and pith tissues, develop during the reproductive stages of the plant [49]. The molecular processes underlying plant defenses against this fungal pathogen are not well-studied. However, induction of systemic acquired resistance by treatments with chemical inducers can increase resistance to the fungus [50], and glyceollin can suppress fungal growth in artificial media, suggesting that isoflavones are part of the soybean defense arsenal against this pathogen [51]. It has been suggested that susceptibility to *C*. *gregata* is associated with the ability of the pathogen to colonize the plant vascular system [49]. We observed that infestation of soybean plants with SCN or BSR fungus did not have any significant effects on the composition of fatty acids in leaves or seeds when applied individually, despite the fact that yield decreases were observed in both cases compared to the no-treatment control. These results suggest that the observed changes in fatty acid levels are aphid specific rather than a general soybean response to pests. However, we cannot discard the possibility that these changes are also triggered by other insects feeding on soybean. Further experiments testing the effect of chewing insects and other phloem-sucking insects that could feed on soybean are necessary to test this possibility.

These results support the hypothesis that soybean aphids are able to suppress effective defense responses. A similar conclusion was achieved in a transcriptome analysis of soybean responses to aphid colonization described previously [18]. The transcriptome analysis showed that, in short term interactions [1 day post-infestation (dpi)], aphids induce genes associated with JA biosynthesis and also JA-responsive genes. However, after longer exposure to aphids (7 dpi), the response to JA is completely suppressed even though JA biosynthetic genes show a 5-fold increase when compared to aphid-infested plants at 1 dpi, and 15-fold more than non-infested plants. Our fatty acid analysis could explain this observation. At 7 dpi, aphids may block JA production by reducing the levels of precursors of the oxylipin pathway. Compensatory mechanisms that sense a deficiency in JA signaling could then increase the expression level of JA biosynthetic genes to increase JA production, albeit unsuccessfully. A study of the effect of SBA infestation on the performance of other pests colonizing soybean plants at the same time indicated that aphids increase soybean susceptibility to SCN, again supporting the hypothesis that SBA are able to suppress defense responses effective against herbivores [30, 31]. Moreover, it has been observed that initial feeding by conspecifics increased the survival of subsequent SBA populations on both susceptible and resistant soybean lines, indicating that SBA can trigger an induced-susceptibility response in soybean [52, 53].

**Fig 6:**
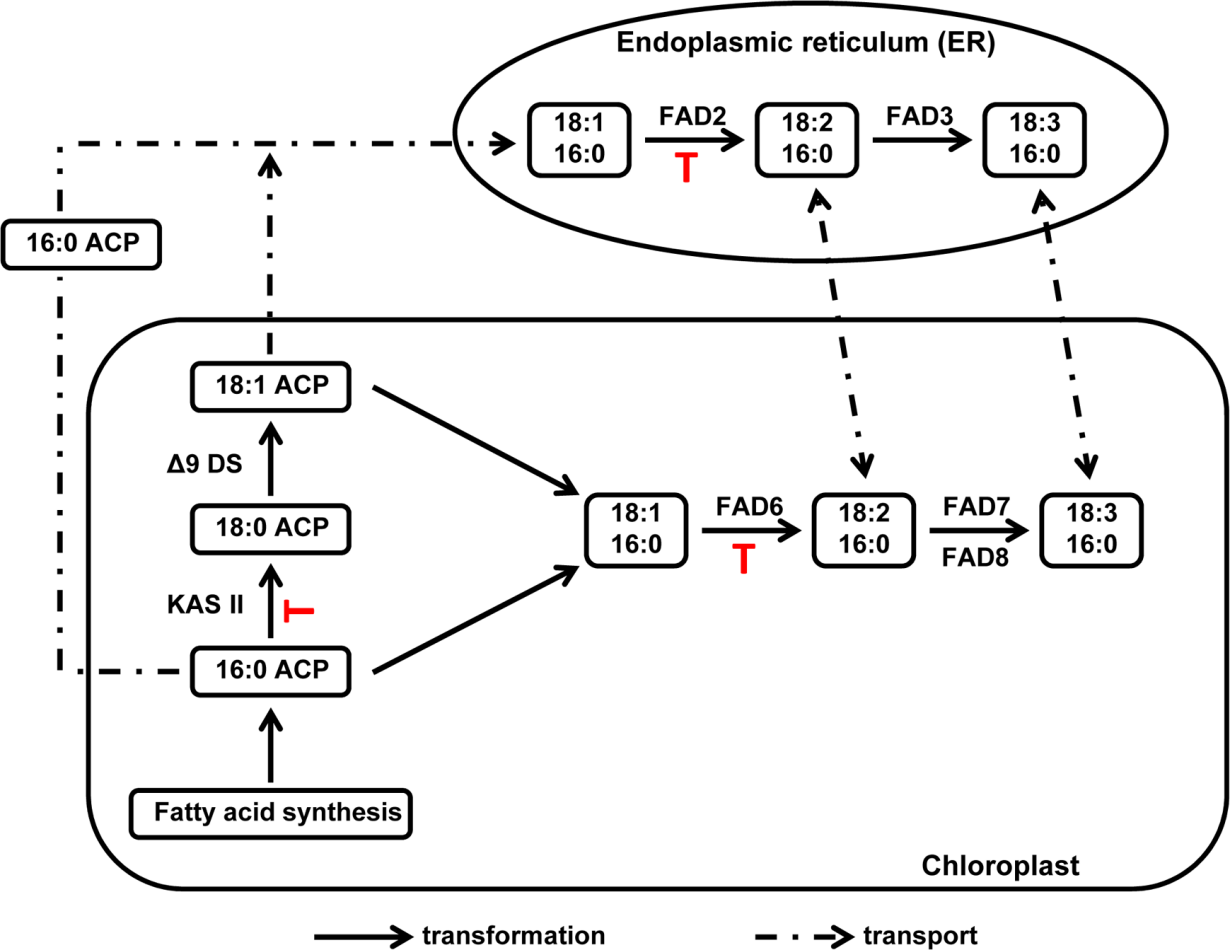
Fatty acid pathway in soybean, and potential points of regulation triggered by aphid infestation. Our working hypothesis suggests that interfering with desaturation by reduction of FAD2 activity in the ER leads to reduction in 18:2 content and consequently 18:3 content in seeds, with a corresponding increase in 18:0 as seen in soybean seeds from aphid-infested plants. In the aphid-infested leaves, we observed increase in 16:0 and reduction in 18:2 and 18:3. These changes could result from a block in FAD6 and negative feedback inhibition of KASII that causes an increase in 16:0 rather than increases in 18:0 and 18:1. Alternatively, KASII and FAD6 could be independently affected by aphid infestation. Putative points of regulation in response to aphid feeding are marked with a red symbol. Only relevant enzymatic reactions and transport events are shown for simplicity.

PUFAs are produced in plants through two parallel pathways (Fig 6). After generation of palmitate (16:0) in the chloroplast, this FA can be further elongated by KAS II to produce stearate (18:0) or directly desaturated to produce 16:1, 16:2 and 16:3 fatty acids. Stearate is also desaturated to produce oleate (18:1) in the chloroplast. Oleate is then desaturated to produce linoleic (18:2) and linolenic (18:3) acids. Oleate desaturation is achieved either through the chloroplast pathway or through a microsomal pathway after oleate is transported to the endoplasmic reticulum (ER) [54, 55]. In some plants, including soybean, the main PUFAs are 18:2 and 18:3, while 16:2 and 16:3 are mostly absent. These plants are normally referred to as “18:3 plants” [54].

How do aphids affect PUFA production? Due to the economic importance of soybean oil, the production of soybean seed PUFAs has been well characterized, and the existence of several mutants in fatty acid accumulation in seeds could provide some clues on this regulation. In soybean seeds, aphid infestation caused a reduction in the amount of linoleic and linolenic acids while stearic and oleic acids increased. Microsomal desaturation is the main contributor of PUFAs in the seed whereas chloroplasts make PUFA mainly in green tissues [56, 57]. Two desaturase activities, an ω-6 fatty acid desaturase (also called fatty acid desaturase 2, FAD2) and an ω-3 fatty acid desaturase (fatty acid desaturase 3, FAD3), are responsible for the conversion from 18:1 to 18:2 and 18:2 to 18:3 respectively, in microsomes. The soybean genome possesses several *FAD2* and *FAD3* genes [58]; and mutants with altered expression of various seed desaturases have been described [59].

*FAD2* mutants that result in “mid-oleic” seed phenotypes show changes similar to those induced by aphids. The mid-oleic soybean line M23 has a mutation in the *FAD2-1a* gene that is expressed in developing embryos [60, 61]. M23 seeds show increases in oleic acid and stearic acid content and reduced levels of linoleic and linolenic acid [62]. On a smaller scale, the same increases in oleic and stearic acids and reduction in the levels of PUFA are observed in seeds of aphid-infested soybean plants. Thus, based on the effect observed in seeds, we propose that aphids affect FAD2 activity, likely the product of the *FAD2-1a* gene, and that some activity may remain because the changes in FA composition are less dramatic than in the *fad2-1a* null mutant.

In contrast, the main source of leaf PUFA is the chloroplast. Chloroplast desaturases have been less well characterized in soybean, and mutant soybean lines lacking these activities are not available, but a large amount of research has focused on Arabidopsis desaturases. The chloroplast enzymes are structurally similar to the microsomal enzymes, and in Arabidopsis are represented by 3 genes: *FAD6*, an oleate desaturase similar to *FAD2*, and *FAD7* and *FAD8*, which are linoleate desaturases similar to *FAD3* [54]. Gene homologs to *FAD6*, *FAD7* and *FAD8* have been found in soybean [58]. Given the structural similarity of the microsomal and chloroplastic fatty acid desaturases, it is possible that any factors that would affect accumulation of PUFAs in the leaves or chloroplasts at a biochemical level will also affect the microsomal fatty acid accumulation; and following the logic presented for the effects observed in seeds, it would be reasonable to propose that aphids affect FAD6 activity in leaves, resulting in a reduction of PUFA in these organs. However, Arabidopsis *fad6* mutants accumulate high levels of oleic acid in leaves in addition to the reduction of PUFA [63] while soybean aphids trigger accumulation of palmitate and decrease of PUFA in soybean leaves. It could be possible that, since soybean is an “18:3 plant”, regulation of PUFA synthesis in soybean chloroplasts is different than in Arabidopsis, and a reduction of FAD6 activity in soybean could result in a negative feedback loop that produces an accumulation of palmitate instead of oleate.

Alternatively, aphids could affect a different step in the production of PUFA. An increase in palmitate levels has been observed in soybean lines with mutations in the *GmKAS IIA* gene [64]. However, the increase in palmitate in these cases is observed in seeds, and it is accompanied by a decrease in oleic acid levels and increase in linoleic acid levels, but not changes in linolenic acid [65]. Since at least two *KAS II* genes exist in the soybean genome [64], it is possible that aphids affect a different KAS II enzyme than the one previously characterized, and the effects in this case could be different. Aphids could also affect the activity of KAS II and FAD2 or FAD6 simultaneously, resulting on the effects described here.

Our working hypothesis is that aphids indirectly affect the levels of PUFAs in soybean by eliciting a plant response that interferes with the desaturation of oleic acid to linoleic and linolenic acids in the chloroplast and microsome, by modulating the activity of FAD2 and FAD6 enzymes, and potentially the elongation of 16:0 to 18:0, through regulation of KAS II activity (Fig 6). However, other mechanisms cannot be overlooked. For example, palmitic and oleic acids are the main FAs that are transported across the plastidic membranes to the ER [55, 56], and regulation of transport could also have an effect on the accumulation of PUFA. Thus, more work is needed to understand this regulation and the effect of reduced PUFA on the defense responses of soybean.

We also found that the effects triggered by aphids on fatty acid metabolism do not persist after aphid removal. The SBA:250 treatment that followed management recommendations to limit aphid colonization did not result in changes in fatty acid levels in our analysis, even though it is known that aphids induce molecular responses in soybean plants when populations are smaller than 250 aphids per plant [17, 18]. Interestingly, it was recently shown that aphid-induced susceptibility is still observed 5 days after removal of the aphids from soybean plants, but no longer observable 9 days after aphid removal [53]. In our experiment, fatty acid analysis was performed on leaves that had been free of aphids for approximately 4 weeks after the initial infestation to 250 aphid per plant followed by insecticide treatment; thus it is possible that aphids affected fatty acid levels but the effect had already dissipated at the time of the analysis.

In summary, we showed that soybean aphid infestation of soybean plants results in a decreased amount of PUFAs both in the leaves and seeds whereas palmitic acid increased in leaves and stearic and oleic acids increased in the seed. Soybean cyst nematode and brown stem rot infections did not result in changes in FA profiles. We hypothesize that aphids interfere with fatty acid biosynthesis and desaturation at any of the points leading to synthesis of stearic acid (KAS II) or desaturation of oleic acid to linoleic acid in the endoplasmic reticulum and chloroplast (FAD2 and FAD6, respectively). The desaturases FAD2 and FAD6 are structurally similar [58]; hence it is possible that they are regulated by similar mechanisms. Reduction in PUFAs is known to affect jasmonic acid production, and the results described here support the hypothesis that aphids are able to block effective defense responses. We are currently working to determine the effect of aphids on jasmonic acid biosynthesis and response genes, and to identify the targets of aphid regulation in the fatty acid biosynthetic pathway.

## Material and Methods

### Experimental setup and design

The experiment was conducted during 2008 and 2009 at the Iowa State University Horticulture Research Station north of Ames, in Story Co. Iowa. A detailed description of the field experiment is reported in McCarville et al. [30]. Briefly, soybean was planted in 28 by 51 cm microplots (10 plants per microplot) kept 152 cm apart in six blocks, and each microplot was individually caged to keep aphids within treatments artificially infested, and keep aphids out of the untreated controls. While there was a total of six commercial soybean varieties in the experiment, a subset of two varieties [DK 27–52 and DK 28–52 (Monsanto Company, St. Louis, MO)] were used in the results presented here. The treatments included two soybean aphid treatments, “SBA: unlimited”, where the aphid population was left to develop freely, reaching densities well exceeding 1,000 plant^-1^; and “SBA: 250”, where the population was left to increase to 250 aphids plant^-1^ and then sprayed with lambda-cyhalothrin (Warrior with Zeon Technology, Syngenta Crop Protection, Greensboro, NC) to simulate the recommendations for soybean aphid management [13]. In both treatments, five aphids were placed on a single plant at the V3 stage (i.e. third trifoliate stage [66]). Once the population on the initial plant reached 50 aphids, infested leaves containing 50 aphids, were clipped to all other plants in the cage. In the third treatment “SCN”, soybean plants were infected with SCN from eggs suspended in 50 ml of water that were applied over the soybean seed at planting. In the fourth treatment “BSR”, the soil was infested with BSR by mixing 40 g of BSR-infested sorghum seed into the soil throughout the plot prior to planting, and a “SBA:SCN:BSR” treatment, which consisted of the combination of the SBA: unlimited, SCN, and BSR treatments. An estimate of aphid density was taken twice a week until populations reached 1,000 on the primary plant in the SBA: unlimited treatment, after which aphid densities were estimated once a week until populations declined for two consecutive weeks. The density of SCN eggs per 100cc of soil was determined at the end of the growing season from a six-core soil sample (19 mm diameter, 15–20 cm in length) per plot. The severity of BSR disease was evaluated at the end of season by splitting stems lengthwise and counting the number of nodes exhibiting discolored pith tissue typical of BSR. Each treatment was replicated 6 times in 2008 and 5 times in 2009. The experiment was set up following a randomized complete block design with six blocks.

### Leaf and seed collection

Leaf samples were collected six weeks after aphid infestation when aphid populations were peaking inside the cages. By this time, the plants were at the R4-R5 growth stage [66] and we harvested the youngest fully expanded leaf for fatty acid extraction. Upon harvesting a selected leaf, aphids were wiped off by hand and discarded, and then the leaf was wrapped in a piece of aluminum foil, numbered and immediately frozen in liquid nitrogen, in the field. After harvesting, the samples were transferred onto dry ice for transportation to the laboratory. In the laboratory, the samples were stored at -80°C until further processing. A sample of harvested seed from each microplot was collected and used for fatty acid analysis.

### Fatty acid extraction and analysis

Leaf and seed samples from individual plants for each treatment (6 replicates for 2008 and 5 replicates for 2009) were finely ground using a mortar and pestle in liquid nitrogen prior to fatty acid extraction. Fatty acid extraction was based on a general method described by Hammond and Fehr [67] and Hammond [68] with a few modifications. Briefly, 200 mg of ground leaf tissue or seed from each replicate were weighed into a 10 ml glass tube and lipids were extracted overnight using 1 ml of hexane. Hexane extraction is effective at recovering lipids from plant tissue, as previously described [69]. After overnight incubation, 200 μl of the oil-hexane mixture was drawn out of the tube into a GC vial. Fatty acid transmethylation was done by addition of 500 μl of sodium methoxide into the glass vial and incubation for two hours. To stop the reaction, 150 μl of water was added to the vial and more hexane was then added to the neck of the vial. The samples were then run on a GC using a SP 2330 capillary column (30m x 0.25 mm x 0.2 micrometer) with flame ionization detection (FID) at the W. M. Keck Metabolomics Research Laboratory at Iowa State University. The GLE-64 reference standards were used in the analysis (Nu-Check-Prep Inc. Elysian, MN). Individual fatty acid content was then given as a percent of the total fatty acids extracted.

### Statistical analysis

Fatty acid data were analyzed using multivariate analysis of variance (MANOVA) (Genstat, VSN international Ltd 2000; PROC GLM; SAS Institute 2001), with a confidence interval of 95%. Individual fatty acids were then analyzed using analysis of variance (ANOVA) with Bonferroni correction to account for multiple comparisons, thus using a confidence interval of 99%. Means comparisons were carried out using Tukey’s range test (confidence interval 95%).

## Supporting Information

S1 Appendix MANOVA analysis statistics for the effect of SBA, SCN and BSR on fatty aphid composition of soybean leaves. (PDF)

S2 Appendix ANOVA and means comparison for the effect of SBA, SCN and BSR on fatty acid composition of soybean leaves. (PDF)

S3 Appendix MANOVA analysis statistics for the effect of SBA, SCN and BSR on fatty aphid composition of soybean seeds. (PDF)

S4 Appendix ANOVA and means comparison for the effect of SBA, SCN and BSR on fatty acid composition of soybean seeds (PDF)

S1 Fig Correlation of seed stearic acid content with levels of PUFAs in 2008 and 2009. (DOCX)
