# Supplementary material for: Soybean Aphid Infestation Induces Changes in Fatty Acid Metabolism in Soybean
Source: PLoS One. 2015 Dec 18;10(12):e0145660. doi: 10.1371/journal.pone.0145660 (PMC4684210; doi:10.1371/journal.pone.0145660)
Supplement: S1 Appendix — (PDF) [file pone.0145660.s001.pdf]

**S1 Appendix:** MANOVA analysis statistics for the effect of SBA, SCN and BSR on fatty aphid composition of soybean leaves

| Partial Correlation Coefficients from the Error SSCP Matrix / Prob > \|r\| | | | | | |
|---|---|---|---|---|---|
| DF = 24 | PALMITIC | STEARIC | OLEIC | LINOLEIC | LINOLENIC |
| PALMITIC | 1.000000 | -0.474412<br>0.0166 | 0.021958<br>0.9170 | -0.558795<br>0.0037 | -0.891089<br><.0001 |
| STEARIC | -0.474412<br>0.0166 | 1.000000 | 0.026526<br>0.8998 | 0.564007<br>0.0033 | 0.198873<br>0.3406 |
| OLEIC | 0.021958<br>0.9170 | 0.026526<br>0.8998 | 1.000000 | -0.099350<br>0.6366 | -0.236337<br>0.2554 |
| LINOLEIC | -0.558795<br>0.0037 | 0.564007<br>0.0033 | -0.099350<br>0.6366 | 1.000000 | 0.196968<br>0.3453 |
| LINOLENIC | -0.891089<br><.0001 | 0.198873<br>0.3406 | -0.236337<br>0.2554 | 0.196968<br>0.3453 | 1.000000 |

| MANOVA Test Criteria and Exact F Statistics for the Hypothesis of No Overall Year Effect<br>H = Type III SSCP Matrix for Year<br>E = Error SSCP Matrix<br><br>S=1 M=1.5 N=9 | | | | | |
|---|---|---|---|---|---|
| Statistic | Value | F Value | Num DF | Den DF | Pr > F |
| Wilks' Lambda | 0.09625237 | 37.56 | 5 | 20 | <.0001 |
| Pillai's Trace | 0.90374763 | 37.56 | 5 | 20 | <.0001 |
| Hotelling-Lawley Trace | 9.38935443 | 37.56 | 5 | 20 | <.0001 |
| Roy's Greatest Root | 9.38935443 | 37.56 | 5 | 20 | <.0001 |

| MANOVA Test Criteria and Exact F Statistics for the Hypothesis of No Overall Variety Effect<br>H = Type III SSCP Matrix for Variety<br>E = Error SSCP Matrix<br><br>S=1 M=1.5 N=9 | | | | | |
|---|---|---|---|---|---|
| Statistic | Value | F Value | Num DF | Den DF | Pr > F |
| Wilks' Lambda | 0.63551108 | 2.29 | 5 | 20 | 0.0841 |
| Pillai's Trace | 0.36448892 | 2.29 | 5 | 20 | 0.0841 |
| Hotelling-Lawley Trace | 0.57353668 | 2.29 | 5 | 20 | 0.0841 |
| Roy's Greatest Root | 0.57353668 | 2.29 | 5 | 20 | 0.0841 |

| MANOVA Test Criteria and Exact F Statistics for the Hypothesis of No Overall Year*Variety Effect<br>H = Type III SSCP Matrix for Year*Variety<br>E = Error SSCP Matrix<br><br>S=1 M=1.5 N=9 | | | | | |
|---|---|---|---|---|---|
| Statistic | Value | F Value | Num DF | Den DF | Pr > F |
| Wilks' Lambda | 0.74195386 | 1.39 | 5 | 20 | 0.2698 |
| Pillai's Trace | 0.25804614 | 1.39 | 5 | 20 | 0.2698 |
| Hotelling-Lawley Trace | 0.34779271 | 1.39 | 5 | 20 | 0.2698 |
| Roy's Greatest Root | 0.34779271 | 1.39 | 5 | 20 | 0.2698 |

| MANOVA Test Criteria and F Approximations for the Hypothesis of No Overall Treatment Effect<br>H = Type III SSCP Matrix for Treatment<br>E = Error SSCP Matrix<br><br>S=5 M=-0.5 N=9 | | | | | |
|---|---|---|---|---|---|
| Statistic | Value | F Value | Num DF | Den DF | Pr > F |
| Wilks' Lambda | 0.10134790 | 2.58 | 25 | 75.799 | 0.0008 |
| Pillai's Trace | 1.38364773 | 1.84 | 25 | 120 | 0.0161 |
| Hotelling-Lawley Trace | 4.90176236 | 3.71 | 25 | 40.48 | 0.0001 |
| Roy's Greatest Root | 4.17607494 | 20.05 | 5 | 24 | <.0001 |
| NOTE: F Statistic for Roy's Greatest Root is an upper bound. | | | | | |

| MANOVA Test Criteria and F Approximations for the Hypothesis of No Overall Year*Treatment Effect<br>H = Type III SSCP Matrix for Year*Treatment<br>E = Error SSCP Matrix<br><br>S=5 M=-0.5 N=9 | | | | | |
|---|---|---|---|---|---|
| Statistic | Value | F Value | Num DF | Den DF | Pr > F |
| Wilks' Lambda | 0.23258822 | 1.46 | 25 | 75.799 | 0.1077 |
| Pillai's Trace | 1.10487724 | 1.36 | 25 | 120 | 0.1380 |
| Hotelling-Lawley Trace | 2.05204084 | 1.55 | 25 | 40.48 | 0.1036 |
| Roy's Greatest Root | 1.38181934 | 6.63 | 5 | 24 | 0.0005 |
| NOTE: F Statistic for Roy's Greatest Root is an upper bound. | | | | | |

| MANOVA Test Criteria and F Approximations for the Hypothesis of No Overall Variety*Treatment Effect<br>H = Type III SSCP Matrix for Variety*Treatment<br>E = Error SSCP Matrix<br><br>S=5 M=-0.5 N=9 | | | | | |
|---|---|---|---|---|---|
| **Statistic** | **Value** | **F Value** | **Num DF** | **Den DF** | **Pr > F** |
| **Wilks' Lambda** | 0.50270750 | 0.62 | 25 | 75.799 | 0.9126 |
| **Pillai's Trace** | 0.60374412 | 0.66 | 25 | 120 | 0.8861 |
| **Hotelling-Lawley Trace** | 0.79084382 | 0.60 | 25 | 40.48 | 0.9116 |
| **Roy's Greatest Root** | 0.43172492 | 2.07 | 5 | 24 | 0.1043 |
| NOTE: F Statistic for Roy's Greatest Root is an upper bound. | | | | | |

| MANOVA Test Criteria and F Approximations for the Hypothesis of No Overall Year*Variety*Treatme Effect<br>H = Type III SSCP Matrix for Year*Variety*Treatme<br>E = Error SSCP Matrix<br><br>S=5 M=-0.5 N=9 | | | | | |
|---|---|---|---|---|---|
| **Statistic** | **Value** | **F Value** | **Num DF** | **Den DF** | **Pr > F** |
| **Wilks' Lambda** | 0.40937009 | 0.82 | 25 | 75.799 | 0.7003 |
| **Pillai's Trace** | 0.75101839 | 0.85 | 25 | 120 | 0.6732 |
| **Hotelling-Lawley Trace** | 1.08003953 | 0.82 | 25 | 40.48 | 0.6987 |
| **Roy's Greatest Root** | 0.59368854 | 2.85 | 5 | 24 | 0.0370 |
| NOTE: F Statistic for Roy's Greatest Root is an upper bound. | | | | | |
