# Supplementary material for: Soybean Aphid Infestation Induces Changes in Fatty Acid Metabolism in Soybean
Source: PLoS One. 2015 Dec 18;10(12):e0145660. doi: 10.1371/journal.pone.0145660 (PMC4684210; doi:10.1371/journal.pone.0145660)
Supplement: S2 Appendix — (PDF) [file pone.0145660.s002.pdf]

S2 Appendix: ANOVA and means comparison for the effect of SBA, SCN and BSR on fatty acid composition of soybean leaves

Leaf Palmitic acid (16:0)

Differences of Treatment Least Squares Means
Adjustment for Multiple Comparisons: Tukey

Treatment	Treatment	Estimate	Standard Error	DF	t Value	Pr > |t|	Adj P
BSR	Control	0.3129	3.6518	41	0.09	0.9321	1.0000
BSR	SBA_250	-1.4417	3.6518	41	-0.39	0.6950	0.9987
BSR	SBA_SCN_BSR	-15.8075	3.6518	41	-4.33	<.0001	0.0012
BSR	SBA_UNL	-22.3304	3.6518	41	-6.11	<.0001	<.0001
BSR	SCN	1.1588	3.6518	41	0.32	0.7526	0.9995
Control	SBA_250	-1.7546	3.6518	41	-0.48	0.6334	0.9966
Control	SBA_SCN_BSR	-16.1204	3.6518	41	-4.41	<.0001	0.0010
Control	SBA_UNL	-22.6433	3.6518	41	-6.20	<.0001	<.0001
Control	SCN	0.8458	3.6518	41	0.23	0.8180	0.9999
SBA_250	SBA_SCN_BSR	-14.3658	3.6518	41	-3.93	0.0003	0.0040
SBA_250	SBA_UNL	-20.8887	3.6518	41	-5.72	<.0001	<.0001
SBA_250	SCN	2.6004	3.6518	41	0.71	0.4804	0.9794
SBA_SCN_BSR	SBA_UNL	-6.5229	3.6518	41	-1.79	0.0815	0.4855
SBA_SCN_BSR	SCN	16.9663	3.6518	41	4.65	<.0001	0.0005
SBA_UNL	SCN	23.4892	3.6518	41	6.43	<.0001	<.0001

Tukey Grouping for Treatment Least Squares Means (Alpha=0.05)

LS-means with the same letter are not significantly different.

Treatment	Estimate	
SBA_UNL	36.1267	A
		A
SBA_SCN_BSR	29.6038	A
SBA_250	15.2379	B
		B
BSR	13.7963	B
		B
Control	13.4833	B
		B
SCN	12.6375	B

Leaf Stearic acid (18:0)

Differences of Treatment Least Squares Means
Adjustment for Multiple Comparisons: Tukey

Treatment	Treatment	Estimate	Standard Error	DF	t Value	Pr > |t|	Adj P
BSR	Control	-0.1092	0.4142	41	-0.26	0.7935	0.9998
BSR	SBA_250	-0.09000	0.4142	41	-0.22	0.8291	0.9999
BSR	SBA_SCN_BSR	0.4333	0.4142	41	1.05	0.3016	0.8993
BSR	SBA_UNL	0.3958	0.4142	41	0.96	0.3449	0.9291
BSR	SCN	0.1217	0.4142	41	0.29	0.7705	0.9997
Control	SBA_250	0.01917	0.4142	41	0.05	0.9633	1.0000
Control	SBA_SCN_BSR	0.5425	0.4142	41	1.31	0.1976	0.7781
Control	SBA_UNL	0.5050	0.4142	41	1.22	0.2298	0.8250
Control	SCN	0.2308	0.4142	41	0.56	0.5804	0.9932
SBA_250	SBA_SCN_BSR	0.5233	0.4142	41	1.26	0.2136	0.8027
SBA_250	SBA_UNL	0.4858	0.4142	41	1.17	0.2476	0.8470
SBA_250	SCN	0.2117	0.4142	41	0.51	0.6121	0.9955
SBA_SCN_BSR	SBA_UNL	-0.03750	0.4142	41	-0.09	0.9283	1.0000
SBA_SCN_BSR	SCN	-0.3117	0.4142	41	-0.75	0.4561	0.9738
SBA_UNL	SCN	-0.2742	0.4142	41	-0.66	0.5118	0.9851

Tukey Grouping for Treatment Least Squares Means (Alpha=0.05)

LS-means with the same letter are not significantly different.

Treatment	Estimate	
Control	6.8371	A
		A
SBA_250	6.8179	A
		A
BSR	6.7279	A
		A
SCN	6.6063	A
		A
SBA_UNL	6.3321	A
		A
SBA_SCN_BSR	6.2946	A

Leaf Oleic Acid

Differences of Treatment Least Squares Means Adjustment for Multiple Comparisons: Tukey							
Treatment	Treatment	Estimate	Standard Error	DF	t Value	Pr > |t|	Adj P
BSR	Control	-0.3446	0.9156	41	-0.38	0.7086	0.9989
BSR	SBA_250	0.7517	0.9156	41	0.82	0.4164	0.9619
BSR	SBA_SCN_BSR	-1.5146	0.9156	41	-1.65	0.1057	0.5687
BSR	SBA_UNL	-0.4608	0.9156	41	-0.50	0.6174	0.9958
BSR	SCN	-0.3525	0.9156	41	-0.39	0.7022	0.9988
Control	SBA_250	1.0963	0.9156	41	1.20	0.2380	0.8356
Control	SBA_SCN_BSR	-1.1700	0.9156	41	-1.28	0.2085	0.7951
Control	SBA_UNL	-0.1163	0.9156	41	-0.13	0.8996	1.0000
Control	SCN	-0.00792	0.9156	41	-0.01	0.9931	1.0000
SBA_250	SBA_SCN_BSR	-2.2663	0.9156	41	-2.48	0.0175	0.1554
SBA_250	SBA_UNL	-1.2125	0.9156	41	-1.32	0.1927	0.7700
SBA_250	SCN	-1.1042	0.9156	41	-1.21	0.2347	0.8314
SBA_SCN_BSR	SBA_UNL	1.0537	0.9156	41	1.15	0.2564	0.8569
SBA_SCN_BSR	SCN	1.1621	0.9156	41	1.27	0.2115	0.7996
SBA_UNL	SCN	0.1083	0.9156	41	0.12	0.9064	1.0000

Tukey Grouping for Treatment Least Squares Means (Alpha=0.05)		
LS-means with the same letter are not significantly different.		
Treatment	Estimate	
SBA_SCN_BSR	6.1075	A
		A
SBA_UNL	5.0538	A
		A
SCN	4.9454	A
		A
Control	4.9375	A
		A
BSR	4.5929	A
		A
SBA_250	3.8413	A

Leaf Linoleic acid

Differences of Treatment Least Squares Means Adjustment for Multiple Comparisons: Tukey							
Treatment	Treatment	Estimate	Standard Error	DF	t Value	Pr > |t|	Adj P
BSR	Control	0.1037	1.4008	41	0.07	0.9413	1.0000
BSR	SBA_250	-1.3387	1.4008	41	-0.96	0.3448	0.9291
BSR	SBA_SCN_BSR	3.6363	1.4008	41	2.60	0.0130	0.1213
BSR	SBA_UNL	5.8717	1.4008	41	4.19	0.0001	0.0019
BSR	SCN	0.4546	1.4008	41	0.32	0.7472	0.9995
Control	SBA_250	-1.4425	1.4008	41	-1.03	0.3091	0.9051
Control	SBA_SCN_BSR	3.5325	1.4008	41	2.52	0.0157	0.1415
Control	SBA_UNL	5.7679	1.4008	41	4.12	0.0002	0.0023
Control	SCN	0.3508	1.4008	41	0.25	0.8035	0.9999
SBA_250	SBA_SCN_BSR	4.9750	1.4008	41	3.55	0.0010	0.0117
SBA_250	SBA_UNL	7.2104	1.4008	41	5.15	<.0001	<.0001
SBA_250	SCN	1.7933	1.4008	41	1.28	0.2077	0.7939
SBA_SCN_BSR	SBA_UNL	2.2354	1.4008	41	1.60	0.1182	0.6059
SBA_SCN_BSR	SCN	-3.1817	1.4008	41	-2.27	0.0284	0.2290
SBA_UNL	SCN	-5.4171	1.4008	41	-3.87	0.0004	0.0049

Tukey Grouping for Treatment Least Squares Means (Alpha=0.05)			
LS-means with the same letter are not significantly different.			
Treatment	Estimate		
SBA_250	26.1733		A
			A
BSR	24.8346	B	A
		B	A
Control	24.7308	B	A
		B	A
SCN	24.3800	B	A
		B	
SBA_SCN_BSR	21.1983	B	C
			C
SBA_UNL	18.9629		C

Leaf Linolenic Acid

Differences of Treatment Least Squares Means Adjustment for Multiple Comparisons: Tukey							
Treatment	Treatment	Estimate	Standard Error	DF	t Value	Pr > |t|	Adj P
BSR	Control	0.07375	2.9790	41	0.02	0.9804	1.0000
BSR	SBA_250	2.1492	2.9790	41	0.72	0.4747	0.9782
BSR	SBA_SCN_BSR	13.2667	2.9790	41	4.45	<.0001	0.0009
BSR	SBA_UNL	16.5338	2.9790	41	5.55	<.0001	<.0001
BSR	SCN	-1.3708	2.9790	41	-0.46	0.6478	0.9972
Control	SBA_250	2.0754	2.9790	41	0.70	0.4899	0.9813
Control	SBA_SCN_BSR	13.1929	2.9790	41	4.43	<.0001	0.0009
Control	SBA_UNL	16.4600	2.9790	41	5.53	<.0001	<.0001
Control	SCN	-1.4446	2.9790	41	-0.48	0.6303	0.9965
SBA_250	SBA_SCN_BSR	11.1175	2.9790	41	3.73	0.0006	0.0071
SBA_250	SBA_UNL	14.3846	2.9790	41	4.83	<.0001	0.0003
SBA_250	SCN	-3.5200	2.9790	41	-1.18	0.2442	0.8430
SBA_SCN_BSR	SBA_UNL	3.2671	2.9790	41	1.10	0.2792	0.8799
SBA_SCN_BSR	SCN	-14.6375	2.9790	41	-4.91	<.0001	0.0002
SBA_UNL	SCN	-17.9046	2.9790	41	-6.01	<.0001	<.0001

Tukey Grouping for Treatment Least Squares Means (Alpha=0.05)		
LS-means with the same letter are not significantly different.		
Treatment	Estimate	
SCN	51.4487	A
		A
BSR	50.0779	A
		A
Control	50.0042	A
		A
SBA_250	47.9288	A
SBA_SCN_BSR	36.8113	B
		B
SBA_UNL	33.5442	B
