# Supplementary material for: Soybean Aphid Infestation Induces Changes in Fatty Acid Metabolism in Soybean
Source: PLoS One. 2015 Dec 18;10(12):e0145660. doi: 10.1371/journal.pone.0145660 (PMC4684210; doi:10.1371/journal.pone.0145660)
Supplement: S3 Appendix — (PDF) [file pone.0145660.s003.pdf]

S3 Appendix: MANOVA analysis statistics for the effect of SBA, SCN and BSR on fatty aphid composition of soybean seeds

Partial Correlation Coefficients from the Error SSCP Matrix / Prob > |r|					
DF = 96	PAL	STE	OLE	LLE	LLN
PAL	1.000000	-0.051791<br>0.6144	-0.304347<br>0.0024	-0.083798<br>0.4145	0.110073<br>0.2831
STE	-0.051791<br>0.6144	1.000000	0.609382<br><.0001	-0.790142<br><.0001	-0.343054<br>0.0006
OLE	-0.304347<br>0.0024	0.609382<br><.0001	1.000000	-0.809471<br><.0001	-0.599459<br><.0001
LLE	-0.083798<br>0.4145	-0.790142<br><.0001	-0.809471<br><.0001	1.000000	0.218646<br>0.0314
LLN	0.110073<br>0.2831	-0.343054<br>0.0006	-0.599459<br><.0001	0.218646<br>0.0314	1.000000

MANOVA Test Criteria and Exact F Statistics for the Hypothesis of No Overall Year Effect<br>H = Type III SSCP Matrix for Year<br>E = Error SSCP Matrix<br><br>S=1 M=1.5 N=45					
Statistic	Value	F Value	Num DF	Den DF	Pr > F
Wilks' Lambda	0.16467160	93.34	5	92	<.0001
Pillai's Trace	0.83532840	93.34	5	92	<.0001
Hotelling-Lawley Trace	5.07269255	93.34	5	92	<.0001
Roy's Greatest Root	5.07269255	93.34	5	92	<.0001

MANOVA Test Criteria and Exact F Statistics for the Hypothesis of No Overall Variety Effect<br>H = Type III SSCP Matrix for Variety<br>E = Error SSCP Matrix<br><br>S=1 M=1.5 N=45					
Statistic	Value	F Value	Num DF	Den DF	Pr > F
Wilks' Lambda	0.14206375	111.12	5	92	<.0001
Pillai's Trace	0.85793625	111.12	5	92	<.0001
Hotelling-Lawley Trace	6.03909355	111.12	5	92	<.0001
Roy's Greatest Root	6.03909355	111.12	5	92	<.0001

MANOVA Test Criteria and Exact F Statistics for the Hypothesis of No Overall Year*Variety Effect<br>H = Type III SSCP Matrix for Year*Variety<br>E = Error SSCP Matrix<br><br>S=1 M=1.5 N=45					
**Statistic**	**Value**	**F Value**	**Num DF**	**Den DF**	**Pr > F**
**Wilks' Lambda**	0.82355970	3.94	5	92	0.0028
**Pillai's Trace**	0.17644030	3.94	5	92	0.0028
**Hotelling-Lawley Trace**	0.21424106	3.94	5	92	0.0028
**Roy's Greatest Root**	0.21424106	3.94	5	92	0.0028

MANOVA Test Criteria and F Approximations for the Hypothesis of No Overall Treatment Effect<br>H = Type III SSCP Matrix for Treatment<br>E = Error SSCP Matrix<br><br>S=5 M=-0.5 N=45					
**Statistic**	**Value**	**F Value**	**Num DF**	**Den DF**	**Pr > F**
**Wilks' Lambda**	0.35026604	4.48	25	343.27	<.0001
**Pillai's Trace**	0.75862736	3.43	25	480	<.0001
**Hotelling-Lawley Trace**	1.55410139	5.65	25	216.6	<.0001
**Roy's Greatest Root**	1.34285198	25.78	5	96	<.0001
NOTE: F Statistic for Roy's Greatest Root is an upper bound.					

MANOVA Test Criteria and F Approximations for the Hypothesis of No Overall Year*Treatment Effect<br>H = Type III SSCP Matrix for Year*Treatment<br>E = Error SSCP Matrix<br><br>S=5 M=-0.5 N=45					
**Statistic**	**Value**	**F Value**	**Num DF**	**Den DF**	**Pr > F**
**Wilks' Lambda**	0.55010596	2.40	25	343.27	0.0003
**Pillai's Trace**	0.51254923	2.19	25	480	0.0009
**Hotelling-Lawley Trace**	0.70748291	2.57	25	216.6	0.0001
**Roy's Greatest Root**	0.51217609	9.83	5	96	<.0001
NOTE: F Statistic for Roy's Greatest Root is an upper bound.					

MANOVA Test Criteria and F Approximations for the Hypothesis of No Overall Variety*Treatment Effect<br>H = Type III SSCP Matrix for Variety*Treatment<br>E = Error SSCP Matrix<br><br>S=5 M=-0.5 N=45					
**Statistic**	**Value**	**F Value**	**Num DF**	**Den DF**	**Pr > F**
**Wilks' Lambda**	0.70028576	1.38	25	343.27	0.1077
**Pillai's Trace**	0.33169150	1.36	25	480	0.1138
**Hotelling-Lawley Trace**	0.38405019	1.40	25	216.6	0.1074
**Roy's Greatest Root**	0.23080725	4.43	5	96	0.0011
**NOTE: F Statistic for Roy's Greatest Root is an upper bound.**					

MANOVA Test Criteria and F Approximations for the Hypothesis of No Overall Year*Variety*Treatme Effect<br>H = Type III SSCP Matrix for Year*Variety*Treatme<br>E = Error SSCP Matrix<br><br>S=5 M=-0.5 N=45					
**Statistic**	**Value**	**F Value**	**Num DF**	**Den DF**	**Pr > F**
**Wilks' Lambda**	0.65161516	1.68	25	343.27	0.0236
**Pillai's Trace**	0.37865918	1.57	25	480	0.0395
**Hotelling-Lawley Trace**	0.48938988	1.78	25	216.6	0.0158
**Roy's Greatest Root**	0.38200831	7.33	5	96	<.0001
**NOTE: F Statistic for Roy's Greatest Root is an upper bound.**					
