# Supplementary material for: Soybean Aphid Infestation Induces Changes in Fatty Acid Metabolism in Soybean
Source: PLoS One. 2015 Dec 18;10(12):e0145660. doi: 10.1371/journal.pone.0145660 (PMC4684210; doi:10.1371/journal.pone.0145660)
Supplement: S4 Appendix — (PDF) [file pone.0145660.s004.pdf]

S4 Appendix: ANOVA and means comparison for the effect of SBA, SCN and BSR on fatty acid composition of soybean seeds

Seed Palmitic acid (16:0)

Type III Tests of Fixed Effects				
Effect	Num DF	Den DF	F Value	Pr > F
Year	1	96	31.62	<.0001
Variety	1	96	310.90	<.0001
Year*Variety	1	96	0.37	0.5440
Treatment	5	96	1.03	0.4050
Year*Treatment	5	96	0.36	0.8725
Variety*Treatment	5	96	0.49	0.7828
Year*Variety*Treatme	5	96	1.18	0.3246

Seed Stearic acid (18:0)

Type III Tests of Fixed Effects				
Effect	Num DF	Den DF	F Value	Pr > F
Year	1	96	73.62	<.0001
Variety	1	96	165.35	<.0001
Year*Variety	1	96	1.64	0.2040
Treatment	5	96	22.34	<.0001
Year*Treatment	5	96	6.50	<.0001
Variety*Treatment	5	96	1.95	0.0931
Year*Variety*Treatme	5	96	3.17	0.0108

Simple Differences of Year*Variety*Treatme Least Squares Means Adjustment for Multiple Comparisons: Tukey								
Slice	Treatment	Treatment	Estimate	Standard Error	DF	t Value	Pr > |t|	Adj P
Year 1 Variety Resistant	BSR	CONTROL	-0.1180	0.2731	96	-0.43	0.6667	0.9980
Year 1 Variety Resistant	BSR	SBA_250	0.1720	0.2731	96	0.63	0.5304	0.9885
Year 1 Variety Resistant	BSR	SBA_SCN_BSR	-0.4480	0.2731	96	-1.64	0.1042	0.5742
Year 1 Variety Resistant	BSR	SBA_UNL	-0.3880	0.2731	96	-1.42	0.1587	0.7145
Year 1 Variety Resistant	BSR	SCN	0.1300	0.2731	96	0.48	0.6352	0.9969
Year 1 Variety Resistant	CONTROL	SBA_250	0.2900	0.2731	96	1.06	0.2910	0.8952
Year 1 Variety Resistant	CONTROL	SBA_SCN_BSR	-0.3300	0.2731	96	-1.21	0.2299	0.8319
Year 1 Variety Resistant	CONTROL	SBA_UNL	-0.2700	0.2731	96	-0.99	0.3254	0.9205
Year 1 Variety Resistant	CONTROL	SCN	0.2480	0.2731	96	0.91	0.3661	0.9436
Year 1 Variety Resistant	SBA_250	SBA_SCN_BSR	-0.6200	0.2731	96	-2.27	0.0254	0.2166
Year 1 Variety Resistant	SBA_250	SBA_UNL	-0.5600	0.2731	96	-2.05	0.0431	0.3223
Year 1 Variety Resistant	SBA_250	SCN	-0.04200	0.2731	96	-0.15	0.8781	1.0000
Year 1 Variety Resistant	SBA_SCN_BSR	SBA_UNL	0.06000	0.2731	96	0.22	0.8266	0.9999
Year 1 Variety Resistant	SBA_SCN_BSR	SCN	0.5780	0.2731	96	2.12	0.0369	0.2878
Year 1 Variety Resistant	SBA_UNL	SCN	0.5180	0.2731	96	1.90	0.0609	0.4106

Tukey Grouping for Year*Variety*Treatme Least Squares Means Slice (Alpha=0.05)			
LS-means with the same letter are not significantly different.			
Slice	Treatment	Estimate	
Year 1 Variety Resistant	SBA_SCN_BSR	5.1480	A
Year 1 Variety Resistant			A
Year 1 Variety Resistant	SBA_UNL	5.0880	A
Year 1 Variety Resistant			A
Year 1 Variety Resistant	CONTROL	4.8180	A
Year 1 Variety Resistant			A
Year 1 Variety Resistant	BSR	4.7000	A
Year 1 Variety Resistant			A
Year 1 Variety Resistant	SCN	4.5700	A
Year 1 Variety Resistant			A
Year 1 Variety Resistant	SBA_250	4.5280	A

Simple Differences of Year*Variety*Treatme Least Squares Means<br>Adjustment for Multiple Comparisons: Tukey								
**Slice**	**Treatment**	**Treatment**	**Estimate**	**Standard Error**	**DF**	**t Value**	**Pr > |t|**	**Adj P**
Year 1 Variety Susceptible	BSR	CONTROL	-0.00800	0.2731	96	-0.03	0.9767	1.0000
Year 1 Variety Susceptible	BSR	SBA_250	-0.1180	0.2731	96	-0.43	0.6667	0.9980
Year 1 Variety Susceptible	BSR	SBA_SCN_BSR	-0.5340	0.2731	96	-1.96	0.0535	0.3757
Year 1 Variety Susceptible	BSR	SBA_UNL	-0.7260	0.2731	96	-2.66	0.0092	0.0935
Year 1 Variety Susceptible	BSR	SCN	-0.1360	0.2731	96	-0.50	0.6197	0.9961
Year 1 Variety Susceptible	CONTROL	SBA_250	-0.1100	0.2731	96	-0.40	0.6880	0.9986
Year 1 Variety Susceptible	CONTROL	SBA_SCN_BSR	-0.5260	0.2731	96	-1.93	0.0571	0.3930
Year 1 Variety Susceptible	CONTROL	SBA_UNL	-0.7180	0.2731	96	-2.63	0.0100	0.1002
Year 1 Variety Susceptible	CONTROL	SCN	-0.1280	0.2731	96	-0.47	0.6404	0.9971
Year 1 Variety Susceptible	SBA_250	SBA_SCN_BSR	-0.4160	0.2731	96	-1.52	0.1310	0.6504
Year 1 Variety Susceptible	SBA_250	SBA_UNL	-0.6080	0.2731	96	-2.23	0.0283	0.2356
Year 1 Variety Susceptible	SBA_250	SCN	-0.01800	0.2731	96	-0.07	0.9476	1.0000
Year 1 Variety Susceptible	SBA_SCN_BSR	SBA_UNL	-0.1920	0.2731	96	-0.70	0.4838	0.9812
Year 1 Variety Susceptible	SBA_SCN_BSR	SCN	0.3980	0.2731	96	1.46	0.1483	0.6920
Year 1 Variety Susceptible	SBA_UNL	SCN	0.5900	0.2731	96	2.16	0.0332	0.2662

Tukey Grouping for Year*Variety*Treatme Least Squares Means Slice (Alpha=0.05)			
LS-means with the same letter are not significantly different.			
**Slice**	**Treatment**	**Estimate**	
Year 1 Variety Susceptible	SBA_UNL	4.3680	A
Year 1 Variety Susceptible			A
Year 1 Variety Susceptible	SBA_SCN_BSR	4.1760	A
Year 1 Variety Susceptible			A
Year 1 Variety Susceptible	SCN	3.7780	A
Year 1 Variety Susceptible			A
Year 1 Variety Susceptible	SBA_250	3.7600	A
Year 1 Variety Susceptible			A
Year 1 Variety Susceptible	CONTROL	3.6500	A
Year 1 Variety Susceptible			A
Year 1 Variety Susceptible	BSR	3.6420	A

Simple Differences of Year*Variety*Treatme Least Squares Means<br>Adjustment for Multiple Comparisons: Tukey								
**Slice**	**Treatment**	**Treatment**	**Estimate**	**Standard Error**	**DF**	**t Value**	**Pr > |t|**	**Adj P**
Year 2 Variety Resistant	BSR	CONTROL	0.2120	0.2731	96	0.78	0.4395	0.9709
Year 2 Variety Resistant	BSR	SBA_250	0.09200	0.2731	96	0.34	0.7370	0.9994
Year 2 Variety Resistant	BSR	SBA_SCN_BSR	-0.1500	0.2731	96	-0.55	0.5841	0.9939
Year 2 Variety Resistant	BSR	SBA_UNL	-2.2540	0.2731	96	-8.25	<.0001	<.0001
Year 2 Variety Resistant	BSR	SCN	0.1800	0.2731	96	0.66	0.5114	0.9859
Year 2 Variety Resistant	CONTROL	SBA_250	-0.1200	0.2731	96	-0.44	0.6614	0.9979
Year 2 Variety Resistant	CONTROL	SBA_SCN_BSR	-0.3620	0.2731	96	-1.33	0.1882	0.7703
Year 2 Variety Resistant	CONTROL	SBA_UNL	-2.4660	0.2731	96	-9.03	<.0001	<.0001
Year 2 Variety Resistant	CONTROL	SCN	-0.03200	0.2731	96	-0.12	0.9070	1.0000
Year 2 Variety Resistant	SBA_250	SBA_SCN_BSR	-0.2420	0.2731	96	-0.89	0.3778	0.9490
Year 2 Variety Resistant	SBA_250	SBA_UNL	-2.3460	0.2731	96	-8.59	<.0001	<.0001
Year 2 Variety Resistant	SBA_250	SCN	0.08800	0.2731	96	0.32	0.7480	0.9995
Year 2 Variety Resistant	SBA_SCN_BSR	SBA_UNL	-2.1040	0.2731	96	-7.70	<.0001	<.0001
Year 2 Variety Resistant	SBA_SCN_BSR	SCN	0.3300	0.2731	96	1.21	0.2299	0.8319
Year 2 Variety Resistant	SBA_UNL	SCN	2.4340	0.2731	96	8.91	<.0001	<.0001

Tukey Grouping for Year*Variety*Treatme Least Squares Means Slice (Alpha=0.05)			
LS-means with the same letter are not significantly different.			
**Slice**	**Treatment**	**Estimate**	
Year 2 Variety Resistant	SBA_UNL	7.5200	A
Year 2 Variety Resistant			
Year 2 Variety Resistant	SBA_SCN_BSR	5.4160	B
Year 2 Variety Resistant			B
Year 2 Variety Resistant	BSR	5.2660	B
Year 2 Variety Resistant			B
Year 2 Variety Resistant	SBA_250	5.1740	B
Year 2 Variety Resistant			B
Year 2 Variety Resistant	SCN	5.0860	B
Year 2 Variety Resistant			B
Year 2 Variety Resistant	CONTROL	5.0540	B

Simple Differences of Year*Variety*Treatme Least Squares Means<br>Adjustment for Multiple Comparisons: Tukey								
**Slice**	**Treatment**	**Treatment**	**Estimate**	**Standard Error**	**DF**	**t Value**	**Pr > |t|**	**Adj P**
Year 2 Variety Susceptible	BSR	CONTROL	0.1340	0.2731	96	0.49	0.6248	0.9964
Year 2 Variety Susceptible	BSR	SBA_250	-0.2360	0.2731	96	-0.86	0.3897	0.9541
Year 2 Variety Susceptible	BSR	SBA_SCN_BSR	-0.4580	0.2731	96	-1.68	0.0968	0.5503
Year 2 Variety Susceptible	BSR	SBA_UNL	-0.9980	0.2731	96	-3.65	0.0004	0.0055
Year 2 Variety Susceptible	BSR	SCN	0.1340	0.2731	96	0.49	0.6248	0.9964
Year 2 Variety Susceptible	CONTROL	SBA_250	-0.3700	0.2731	96	-1.35	0.1787	0.7536
Year 2 Variety Susceptible	CONTROL	SBA_SCN_BSR	-0.5920	0.2731	96	-2.17	0.0327	0.2626
Year 2 Variety Susceptible	CONTROL	SBA_UNL	-1.1320	0.2731	96	-4.14	<.0001	0.0010
Year 2 Variety Susceptible	CONTROL	SCN	-432E-16	0.2731	96	-0.00	1.0000	1.0000
Year 2 Variety Susceptible	SBA_250	SBA_SCN_BSR	-0.2220	0.2731	96	-0.81	0.4183	0.9645
Year 2 Variety Susceptible	SBA_250	SBA_UNL	-0.7620	0.2731	96	-2.79	0.0064	0.0678
Year 2 Variety Susceptible	SBA_250	SCN	0.3700	0.2731	96	1.35	0.1787	0.7536
Year 2 Variety Susceptible	SBA_SCN_BSR	SBA_UNL	-0.5400	0.2731	96	-1.98	0.0509	0.3630
Year 2 Variety Susceptible	SBA_SCN_BSR	SCN	0.5920	0.2731	96	2.17	0.0327	0.2626
Year 2 Variety Susceptible	SBA_UNL	SCN	1.1320	0.2731	96	4.14	<.0001	0.0010

Tukey Grouping for Year*Variety*Treatme Least Squares Means Slice (Alpha=0.05)				
LS-means with the same letter are not significantly different.				
**Slice**	**Treatment**	**Estimate**		
Year 2 Variety Susceptible	SBA_UNL	5.2320		A
Year 2 Variety Susceptible				A
Year 2 Variety Susceptible	SBA_SCN_BSR	4.6920	B	A
Year 2 Variety Susceptible			B	A
Year 2 Variety Susceptible	SBA_250	4.4700	B	A
Year 2 Variety Susceptible			B	
Year 2 Variety Susceptible	BSR	4.2340	B	
Year 2 Variety Susceptible			B	
Year 2 Variety Susceptible	SCN	4.1000	B	
Year 2 Variety Susceptible			B	
Year 2 Variety Susceptible	CONTROL	4.1000	B	

Seed Oleic acid (18:1)

Type III Tests of Fixed Effects				
Effect	Num DF	Den DF	F Value	Pr > F
Year	1	96	143.52	<.0001
Variety	1	96	3.18	0.0775
Year*Variety	1	96	1.87	0.1743
Treatment	5	96	7.28	<.0001
Year*Treatment	5	96	2.33	0.0481
Variety*Treatment	5	96	0.61	0.6924
Year*Variety*Treatme	5	96	2.24	0.0567

Differences of Treatment Least Squares Means Adjustment for Multiple Comparisons: Tukey							
Treatment	Treatment	Estimate	Standard Error	DF	t Value	Pr > |t|	Adj P
BSR	CONTROL	0.2365	0.3358	96	0.70	0.4830	0.9810
BSR	SBA_250	0.1445	0.3358	96	0.43	0.6679	0.9981
BSR	SBA_SCN_BSR	-0.5175	0.3358	96	-1.54	0.1266	0.6388
BSR	SBA_UNL	-1.4705	0.3358	96	-4.38	<.0001	0.0004
BSR	SCN	-0.04900	0.3358	96	-0.15	0.8843	1.0000
CONTROL	SBA_250	-0.09200	0.3358	96	-0.27	0.7847	0.9998
CONTROL	SBA_SCN_BSR	-0.7540	0.3358	96	-2.25	0.0270	0.2272
CONTROL	SBA_UNL	-1.7070	0.3358	96	-5.08	<.0001	<.0001
CONTROL	SCN	-0.2855	0.3358	96	-0.85	0.3973	0.9571
SBA_250	SBA_SCN_BSR	-0.6620	0.3358	96	-1.97	0.0516	0.3664
SBA_250	SBA_UNL	-1.6150	0.3358	96	-4.81	<.0001	<.0001
SBA_250	SCN	-0.1935	0.3358	96	-0.58	0.5658	0.9924
SBA_SCN_BSR	SBA_UNL	-0.9530	0.3358	96	-2.84	0.0055	0.0600
SBA_SCN_BSR	SCN	0.4685	0.3358	96	1.40	0.1662	0.7299
SBA_UNL	SCN	1.4215	0.3358	96	4.23	<.0001	0.0007

Tukey Grouping for Treatment Least Squares Means (Alpha=0.05)			
LS-means with the same letter are not significantly different.			
Treatment	Estimate		
SBA_UNL	22.4235		A
			A
SBA_SCN_BSR	21.4705	B	A
		B	
SCN	21.0020	B	
		B	
BSR	20.9530	B	
		B	
SBA_250	20.8085	B	
		B	
CONTROL	20.7165	B	

Seed Linoleic acid (18:2)

Type III Tests of Fixed Effects				
Effect	Num DF	Den DF	F Value	Pr > F
Year	1	96	10.26	0.0018
Variety	1	96	220.25	<.0001
Year*Variety	1	96	1.91	0.1707
Treatment	5	96	10.83	<.0001
Year*Treatment	5	96	2.72	0.0240
Variety*Treatment	5	96	1.38	0.2393
Year*Variety*Treatme	5	96	2.53	0.0342

Differences of Treatment Least Squares Means Adjustment for Multiple Comparisons: Tukey							
Treatment	Treatment	Estimate	Standard Error	DF	t Value	Pr > |t|	Adj P
BSR	CONTROL	-0.1095	0.3557	96	-0.31	0.7589	0.9996
BSR	SBA_250	-0.08800	0.3557	96	-0.25	0.8051	0.9999
BSR	SBA_SCN_BSR	0.5470	0.3557	96	1.54	0.1274	0.6409
BSR	SBA_UNL	2.0135	0.3557	96	5.66	<.0001	<.0001
BSR	SCN	0.02050	0.3557	96	0.06	0.9542	1.0000
CONTROL	SBA_250	0.02150	0.3557	96	0.06	0.9519	1.0000
CONTROL	SBA_SCN_BSR	0.6565	0.3557	96	1.85	0.0680	0.4419
CONTROL	SBA_UNL	2.1230	0.3557	96	5.97	<.0001	<.0001
CONTROL	SCN	0.1300	0.3557	96	0.37	0.7156	0.9991
SBA_250	SBA_SCN_BSR	0.6350	0.3557	96	1.79	0.0774	0.4801
SBA_250	SBA_UNL	2.1015	0.3557	96	5.91	<.0001	<.0001
SBA_250	SCN	0.1085	0.3557	96	0.31	0.7610	0.9996
SBA_SCN_BSR	SBA_UNL	1.4665	0.3557	96	4.12	<.0001	0.0011
SBA_SCN_BSR	SCN	-0.5265	0.3557	96	-1.48	0.1421	0.6776
SBA_UNL	SCN	-1.9930	0.3557	96	-5.60	<.0001	<.0001

Tukey Grouping for Treatment Least Squares Means (Alpha=0.05)		
LS-means with the same letter are not significantly different.		
Treatment	Estimate	
CONTROL	56.7095	A
		A
SBA_250	56.6880	A
		A
BSR	56.6000	A
		A
SCN	56.5795	A
		A
SBA_SCN_BSR	56.0530	A
SBA_UNL	54.5865	B

Seed Linolenic acid (18:3)

Type III Tests of Fixed Effects				
Effect	Num DF	Den DF	F Value	Pr > F
Year	1	96	461.47	<.0001
Variety	1	96	24.35	<.0001
Year*Variety	1	96	3.18	0.0779
Treatment	5	96	9.42	<.0001
Year*Treatment	5	96	0.75	0.5897
Variety*Treatment	5	96	0.75	0.5855
Year*Variety*Treatme	5	96	0.57	0.7219

Differences of Treatment Least Squares Means Adjustment for Multiple Comparisons: Tukey							
Treatment	Treatment	Estimate	Standard Error	DF	t Value	Pr > |t|	Adj P
BSR	CONTROL	-0.1165	0.1569	96	-0.74	0.4597	0.9761
BSR	SBA_250	-0.1205	0.1569	96	-0.77	0.4445	0.9722
BSR	SBA_SCN_BSR	0.5205	0.1569	96	3.32	0.0013	0.0158
BSR	SBA_UNL	0.6110	0.1569	96	3.89	0.0002	0.0025
BSR	SCN	-0.1060	0.1569	96	-0.68	0.5010	0.9843
CONTROL	SBA_250	-0.00400	0.1569	96	-0.03	0.9797	1.0000
CONTROL	SBA_SCN_BSR	0.6370	0.1569	96	4.06	0.0001	0.0014
CONTROL	SBA_UNL	0.7275	0.1569	96	4.64	<.0001	0.0002
CONTROL	SCN	0.01050	0.1569	96	0.07	0.9468	1.0000
SBA_250	SBA_SCN_BSR	0.6410	0.1569	96	4.08	<.0001	0.0013
SBA_250	SBA_UNL	0.7315	0.1569	96	4.66	<.0001	0.0001
SBA_250	SCN	0.01450	0.1569	96	0.09	0.9266	1.0000
SBA_SCN_BSR	SBA_UNL	0.09050	0.1569	96	0.58	0.5655	0.9923
SBA_SCN_BSR	SCN	-0.6265	0.1569	96	-3.99	0.0001	0.0017
SBA_UNL	SCN	-0.7170	0.1569	96	-4.57	<.0001	0.0002

Tukey Grouping for Treatment Least Squares Means (Alpha=0.05)		
LS-means with the same letter are not significantly different.		
Treatment	Estimate	
SBA_250	8.3180	A
		A
CONTROL	8.3140	A
		A
SCN	8.3035	A
		A
BSR	8.1975	A
SBA_SCN_BSR	7.6770	B
		B
SBA_UNL	7.5865	B
