# Supplementary material for: Soybean Aphid Infestation Induces Changes in Fatty Acid Metabolism in Soybean
Source: PLoS One. 2015 Dec 18;10(12):e0145660. doi: 10.1371/journal.pone.0145660 (PMC4684210; doi:10.1371/journal.pone.0145660)
Supplement: S1 Fig — (DOCX) [file pone.0145660.s005.docx]

## S1 Fig.: Correlation of seed stearic acid content with levels of PUFAs in 2008 and 2009


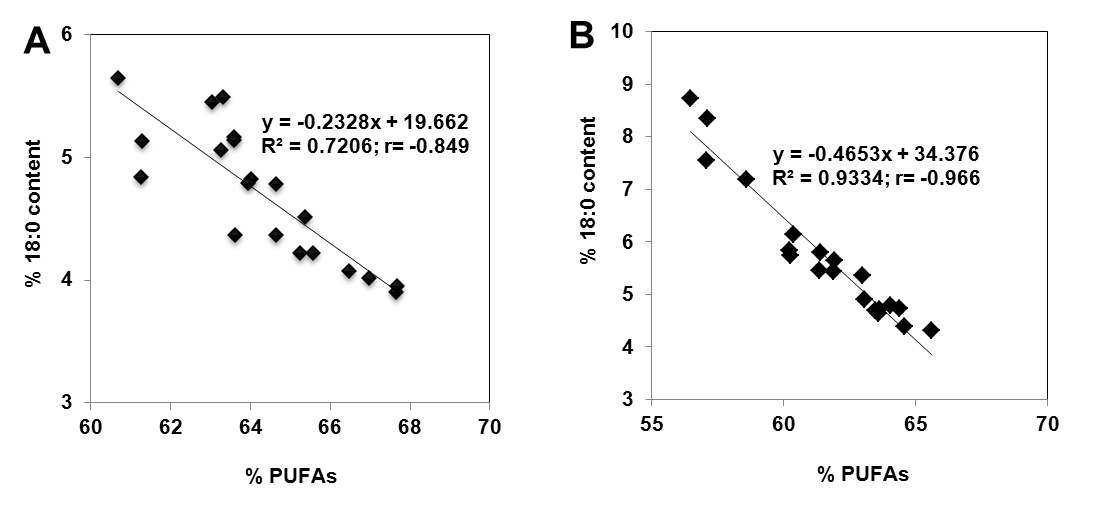


A correlation of seed stearic acid (18:0) content with the content of polyunsaturated fatty acids (18:2 and 18:3, PUFAs) in 2008 (A) and 2009 (B) revealed a strong and negative relationship with coefficients of -0.849 and -0.966, respectively.
